# A fungi-derived cyclic peptide enhances Th9-mediated antitumor immunity by targeting ZAP70 and SREBP1

**DOI:** 10.1172/JCI196907

**Published:** 2025-12-09

**Authors:** Wenli Zhao, Yang Zhou, Yuyang Chen, Yicheng Sun, Jiaxin Tang, Yihan Zhu, Jie Ren, Tianxu Du, Handuo Wang, Yuan Gao, Yu Hu, Ling Jiang, Tomohiko Ohwada, Qi Luo, Enguang Bi

**Affiliations:** 1 Department of Biochemistry and Molecular Biology, School of Basic Medical Sciences, and; 2 Department of Urology, Zhujiang Hospital, Southern Medical University, Guangzhou, Guangdong, China.; 3 Division of Nephrology, Nanfang Hospital, Southern Medical University, State Key Laboratory of Multi-organ Injury Prevention and Treatment, National Clinical Research Center for Kidney Disease, Guangdong Provincial Institute of Nephrology, Guangdong Provincial Key Laboratory of Renal Failure Research, Guangzhou, Guangdong, China.; 4 Department of Hematology, Nanfang Hospital, and; 5 School of Traditional Chinese Medicine, Southern Medical University, Guangzhou, China.; 6 Guangdong Basic Research Center of Excellence for Integrated Traditional and Western Medicine for Qingzhi Diseases, Guangzhou, Guangdong, China.; 7 Guangdong Provincial Key Laboratory of Chinese Medicine Pharmaceutics, School of Traditional Chinese Medicine, Southern Medical University, Guangzhou, Guangdong, China.

**Keywords:** Immunology, Oncology, Cancer immunotherapy, Peptides, T cells

## Abstract

Adoptive cell therapy (ACT) relies on durable and functional T cells to mediate tumor clearance. Th9 cells are a metabolically fit CD4^+^ T cell subset with strong persistence but limited cytotoxicity. Here, we identified endomelipeptide A (EpA), a cyclic peptide isolated from *Ganoderma lucidum*–associated endophytic fungi, as a potent enhancer of Th9 cell differentiation. EpA promoted a cytotoxic Th9 phenotype with enhanced mitochondrial function and metabolic fitness. Mechanistically, EpA dually targeted ZAP70 and SREBP1, coupling T cell receptor signaling activation with lipid metabolism suppression. EpA-treated Th9 cells mediated robust, CD8^+^ T cell–dependent tumor control and enhanced the efficacy of human Th9 CAR T cell therapy in vivo. These findings establish EpA as a distinct cyclic peptide that reprograms Th9 cells and provides a potential approach to boost ACT efficacy.

## Abstract 2

We identify a natural compound that strengthens cancer-fighting immune cells by rewiring their metabolism and signaling, improving the effectiveness of cellular immunotherapy.

## Graphical abstract

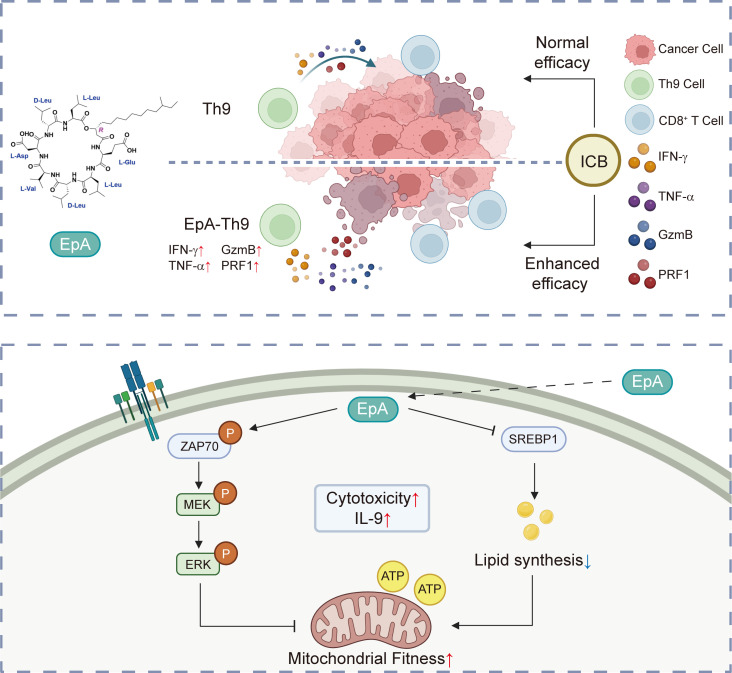

## Introduction

Adoptive cell therapy (ACT) has revolutionized cancer immunotherapy, particularly in hematological malignancies, by harnessing genetically modified or ex vivo–expanded T cells to eliminate tumor cells (1–4). While CD8^+^ cytotoxic T lymphocytes (CTLs) have been the primary focus in ACT development, accumulating evidence highlights the indispensable roles of CD4^+^ T cell subsets in orchestrating effective and durable antitumor responses. CD4^+^ T cells can provide critical help to CD8^+^ T cells, enhance antigen presentation, and, in some cases, exert direct cytotoxic effects (5–9).

Among CD4^+^ subsets, Th1 cells are known for their ability to mediate type I immune responses and license CD8^+^ CTLs through IFN-γ, TNF-α and IL-2 production, DC activation, and chemokine release. However, Th1 cells are prone to exhaustion and apoptosis under tumor-induced metabolic and immunologic stress, limiting their persistence and therapeutic impact (10). Thus, identifying alternative CD4^+^ T cell subsets that couple potent effector activity with long-term durability remains a key goal in ACT research.

Th9 cells have recently emerged as a promising candidate in this context. Defined by their high IL-9 production, Th9 cells exhibit superior persistence, resistance to exhaustion, and stem-like transcriptional signatures (11–13). These traits confer robust and durable antitumor responses in preclinical models. However, despite these advantages, Th9 cells possess limited intrinsic cytotoxicity and have been largely characterized as CD8-independent effectors. Although Th9 cells can transition toward IFN-γ–producing Th1-like states in vivo, their ability to support CD8^+^ T cell activation remains suboptimal (14–16). Enhancing the cytotoxicity and CD8-cooperative capacity of Th9 cells could therefore greatly broaden their clinical utility.

Natural products have long served as a cornerstone of drug discovery and immunomodulation, providing structurally diverse scaffolds with unique bioactivities (17). Among these, cyclic peptides represent a particularly attractive class of molecules owing to their conformational rigidity, proteolytic stability, and ability to modulate intracellular protein-protein interactions (18). Classical examples such as cyclosporine A and FK506 are widely used immunosuppressants, while more recent efforts have repurposed synthetic cyclic peptides to target immune checkpoints, including PD-1, CTLA-4, and LAG-3, for cancer immunotherapy (18–26). However, the potential of natural cyclic peptides to actively enhance T cell differentiation and function, particularly in the setting of ACT, remains largely unexplored.

Endophytic fungi, microorganisms that reside symbiotically within insects, medicinal plants, or fungi, have emerged as prolific producers of bioactive secondary metabolites. These endophytes are not only readily culturable under defined laboratory conditions, but also frequently yield compounds with potent immunoregulatory activities that rival or exceed those of their hosts (27, 28). In our previous work, we demonstrated that small molecules, such as polyketides and 3-methoxy-4′′-deoxyterprenin, isolated from the endophytic *Aspergillus taichungensis* derived from *Periplaneta americana*, can modulate CD4^+^ T cell differentiation, highlighting the untapped immunotherapeutic potential of endophyte-derived compounds (29, 30).

In this study, we identified endomelipeptide A (EpA), a functionally unstudied cyclic peptide isolated from *Ganoderma lucidum*–associated endophytic fungi, as a potent and selective enhancer of Th9 cell differentiation. EpA promoted a cytotoxic Th9 phenotype, improved mitochondrial function, and enhanced metabolic fitness. Mechanistically, EpA acted via dual targeting of ZAP70 and SREBP1, coupling T cell receptor (TCR) signaling amplification with suppression of fatty acid biosynthesis. Functionally, EpA-treated Th9 cells elicited strong CD8^+^ T cell–dependent antitumor responses and significantly improved the efficacy of human Th9 CAR T cell therapy in preclinical models. These findings highlight EpA as a promising immunomodulatory compound for improving T cell–based cancer immunotherapy.

## Results

### Identification of EpA as a potent enhancer of Th9 cell differentiation.

**Figure 1:**
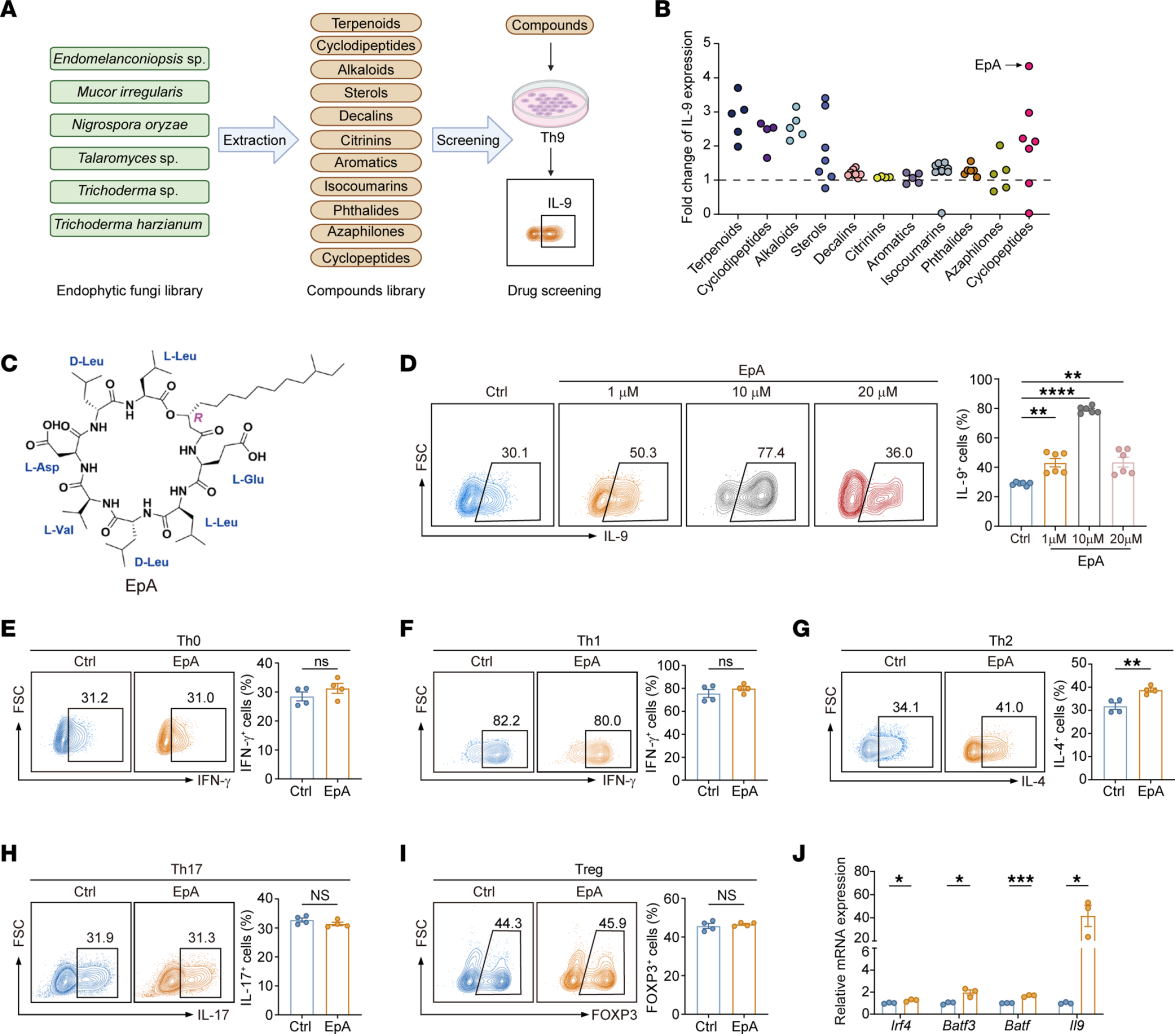
Identification of EpA as a potent and selective enhancer of Th9 cell differentiation. (**A**) Schematic overview of the natural product screening pipeline used to identify small molecules that promote Th9 polarization. (**B**) Scatter plot showing fold changes in IL-9 production by Th9 cells treated with 65 structurally distinct natural compounds (10 μM) compared with the DMSO control. (**C**) Chemical structure of EpA, a cyclic peptide isolated from *Endomelanconiopsis sp*. (**D**) Dose-response analysis of EpA on IL-9^+^ Th9 cell differentiation. Naive CD4^+^ T cells were cultured under Th9-polarizing conditions and analyzed by flow cytometry; representative plots and quantification are shown (*n* = 6). (**E**–**I**) Flow cytometric analysis of CD4^+^ T cells cultured under Th0- (**E**), Th1- (**F**), Th2- (**G**), Th17- (**H**), and Treg-polarizing (**I**) conditions, with or without EpA. Representative plots (left) and quantification (right) of cytokine or Foxp3 expression are shown (*n* = 4). (**J**) Quantitative RT-PCR analysis of Th9 signature genes (*Irf4*, *Batf3*, *Batf*, and *Il9*) in control or EpA-treated Th9 cells (*n* = 3). Statistical analysis was performed using 1-way ANOVA with Tukey’s post hoc test (**D**), 2-tailed unpaired Student’s *t* test (**E**–**J**). Data are presented as the mean ± SEM. *P < 0.05, **P < 0.01, ***P < 0.001, ****P < 0.0001.

To identify natural small molecules that enhance the antitumor potential of Th9 cells, we constructed a chemically and structurally defined small-molecule library derived from endophytic fungi isolated from *G*. *lucidum* and *Periplaneta americana* (Supplemental Table 1; supplemental material available online with this article; https://doi.org/10.1172/JCI196907DS1). This library comprised 65 structurally diverse metabolites classified into 11 distinct scaffold families, with each compound structurally elucidated via NMR spectroscopy and verified against previously reported spectral data. To functionally evaluate the immunomodulatory potential of these compounds, we systematically screened the library for their ability to promote Th9 cell differentiation, assessed by intracellular IL-9 expression using flow cytometry (Figure 1, A and B). Among the screened candidates, a previously reported but functionally uncharacterized cyclolipopeptide — anteiso-C15 Leu_7_ surfactin (31), herein referred to as EpA — emerged as the most potent enhancer of Th9 polarization (Supplemental Table 2 and Figure 1, B and C). EpA robustly induced IL-9 expression and was therefore selected for subsequent mechanistic and functional studies.

To determine the optimal dose, naive CD4^+^ T cells were cultured under Th9-polarizing conditions with increasing concentrations of EpA. IL-9 expression peaked at 10 μM, which was used for all subsequent experiments (Figure 1D). As shown in Figure 1, E–I, EpA did not affect the polarization of Th0 cell, Th1 cell, Th17 cell, or Treg subsets, while inducing only a minor increase in Th2 differentiation, indicating a selective immunomodulatory activity toward the Th9 subset.

In addition to IL-9, EpA treatment significantly upregulated key Th9-associated transcription factors and effector cytokines at the transcriptomic level (Figure 1J). These data demonstrated that EpA specifically enhances Th9 cell differentiation.

### EpA confers superior antitumor efficacy to Th9 cells in a CD8^+^ T cell–dependent manner.

**Figure 2:**
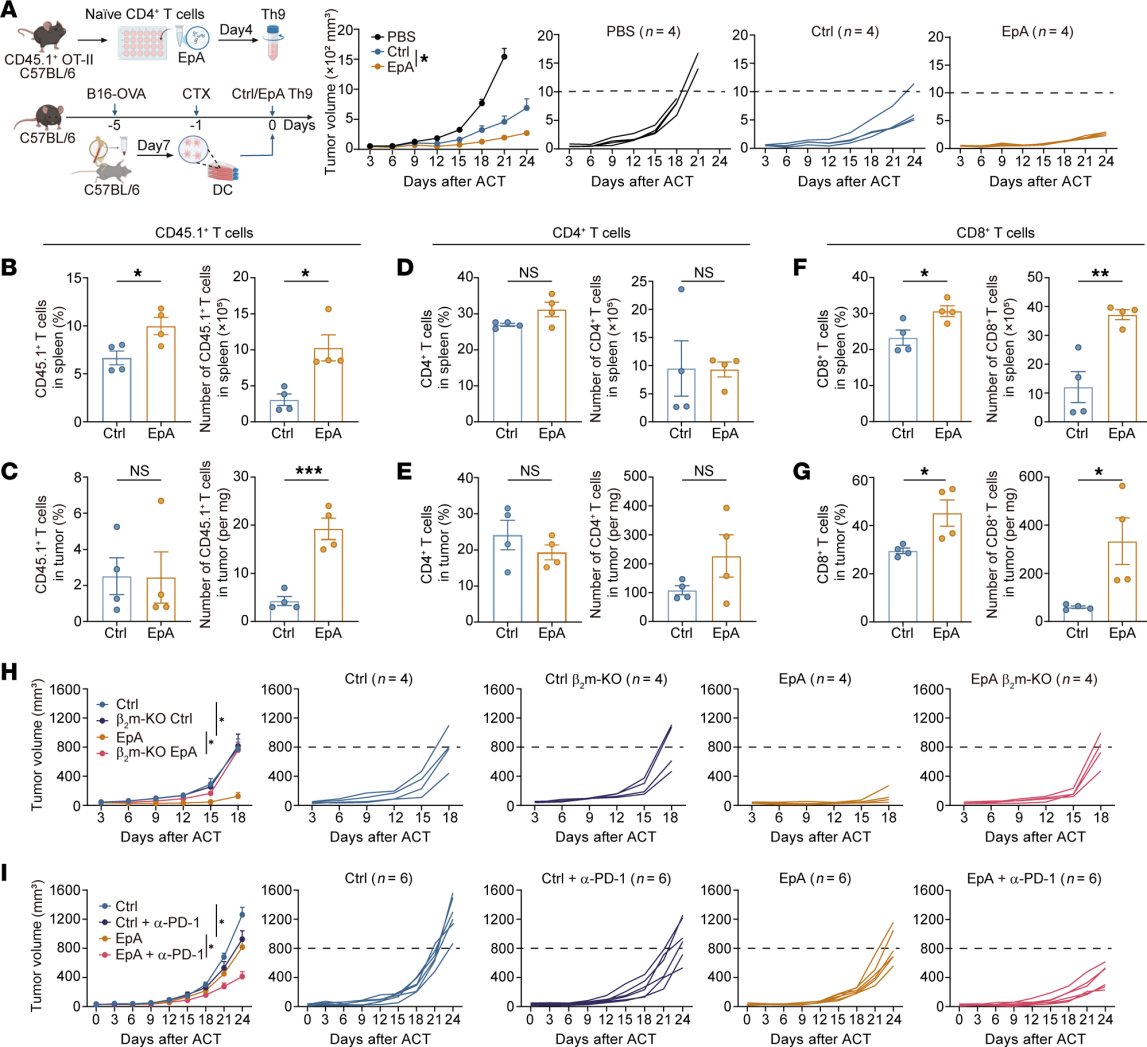
EpA enhances the antitumor activity of Th9 cells in a CD8^+^ T cell–dependent manner. (**A**) Schematic of the therapeutic model. C57BL/6 mice were subcutaneously inoculated with B16-OVA melanoma cells (5 × 10^5^) and preconditioned with cyclophosphamide (CTX) on day 4. On day 5, mice received PBS, OT-II Th9 cells (control), or EpA-treated OT-II Th9 cells (EpA) (2 × 10^6^, i.v.) (*n* = 4 mice per group). (**B**–**G**) Flow-cytometric analysis of transferred CD45.1^+^ OT-II Th9 cells (**B** and **C**) and endogenous CD4^+^ (**D** and **E**) and CD8^+^ T cells (**F** and **G**) in spleens and tumors, showing frequencies and absolute numbers (*n* = 4 mice per group). (**H**) Tumor growth curves in B16-OVA–bearing C57BL/6 or β2-microglobulin–deficient (β_2_m-KO) mice following transfer of control or EpA-treated OT-II Th9 cells (*n* = 4 mice per group). (**I**) C57BL/6 mice bearing B16-OVA tumors were treated with control or EpA-induced OT-II Th9 cells (2 × 10^6^, i.v., day 5), followed by anti–PD-1 antibody (200 μg, i.p.) on day 7, and (100 μg, i.p.) on day 10 and 12. Tumor growth is shown with individual trajectories (*n* = 6 mice per group). Statistical analysis was performed using 2-tailed unpaired Student’s *t* test (**A**, **B**–**G**) or 1-way ANOVA with Tukey’s post hoc test (**H** and **I**). All data are presented as the mean ± SEM. *P < 0.05, **P < 0.01, ***P < 0.001. Schematic in **A** created using BioRender (https://Biorender.com).

To evaluate the antitumor efficacy of EpA-induced Th9 (EpA-Th9) cells, we employed the B16-OVA melanoma model in C57BL/6 mice. CD45.1^+^ OT-II Th9 cells, differentiated with or without EpA, were adoptively transferred into tumor-bearing hosts. Mice receiving EpA-Th9 cells exhibited significantly improved tumor control compared with those treated with conventional Th9 cells (Figure 2A). No significant differences in body weight or serum IL-6 levels were observed among control and treatment groups (Supplemental Figure 1, A and B), indicating that EpA-Th9 therapy was well tolerated. Following transfer, EpA-Th9 cells expanded robustly in the spleen and moderately in the lymph nodes (Figure 2B and Supplemental Figure 1, C and D). Within tumors, although the frequency of transferred cells remained comparable, their absolute numbers were markedly increased (Figure 2C and Supplemental Figure 1E), suggesting that EpA-Th9 cells may undergo peripheral expansion before infiltrating the tumor microenvironment.

To determine whether EpA-Th9 cells influence endogenous immune responses, we analyzed host T cell compartments across lymphoid organs and tumors. The frequencies and absolute numbers of endogenous CD4^+^ T cells showed minimal changes in the spleens, lymph nodes, and tumors (Figure 2, D and E, and Supplemental Figure 1, F–H). In contrast, endogenous CD8^+^ T cells were markedly increased in both frequency and absolute number within the spleens and tumors of EpA-Th9–treated mice (Figure 2, F and G), with a modest frequency increase in lymph nodes despite unchanged total numbers (Supplemental Figure 1I). Moreover, DC frequencies and total counts were significantly elevated in both spleens and lymph nodes of EpA-Th9–treated mice, whereas NK cells increased only in the spleen (Supplemental Figure 1, J–M).

To directly assess the requirement for endogenous CD8^+^ T cells, we utilized β_2_-microglobulin–deficient (β_2_m-KO) mice, which lack functional MHC class I and are therefore devoid of CD8^+^ T cells (32). In this context, the antitumor activity of conventional Th9 cells was preserved, consistent with previous findings that their therapeutic effects are largely CD8^+^ T cell independent (Figure 2H and Supplemental Figure 2A) (15). In contrast, the enhanced antitumor efficacy conferred by EpA-Th9 cells was completely abolished in β_2_m-KO hosts, rendering their effect comparable to that of conventional Th9 cells (Figure 2H). These results demonstrate that the superior tumor control mediated by EpA-Th9 cells is critically dependent on the presence of functional CD8^+^ T cells.

Finally, we examined whether the CD8^+^ T cell–supporting capacity of EpA-Th9 cells could synergize with immune checkpoint blockade (ICB) therapy. B16-OVA tumor-bearing mice were treated with Th9 or EpA-Th9 cells in combination with anti–PD-1 antibody (Supplemental Figure 2B). While conventional Th9 cells conferred no additional benefit in this context, EpA-Th9 cells significantly enhanced the therapeutic response to PD-1 blockade (Figure 2I), further underscoring their capacity to potentiate CD8^+^ T cell–mediated immunity.

Collectively, these data demonstrate that EpA enhances the antitumor activity of Th9 cells by promoting their persistence and licensing of endogenous CD8^+^ T cells.

### EpA promotes the differentiation of cytotoxic Th9 cells with enhanced Th1-like transition in vivo.

**Figure 3:**
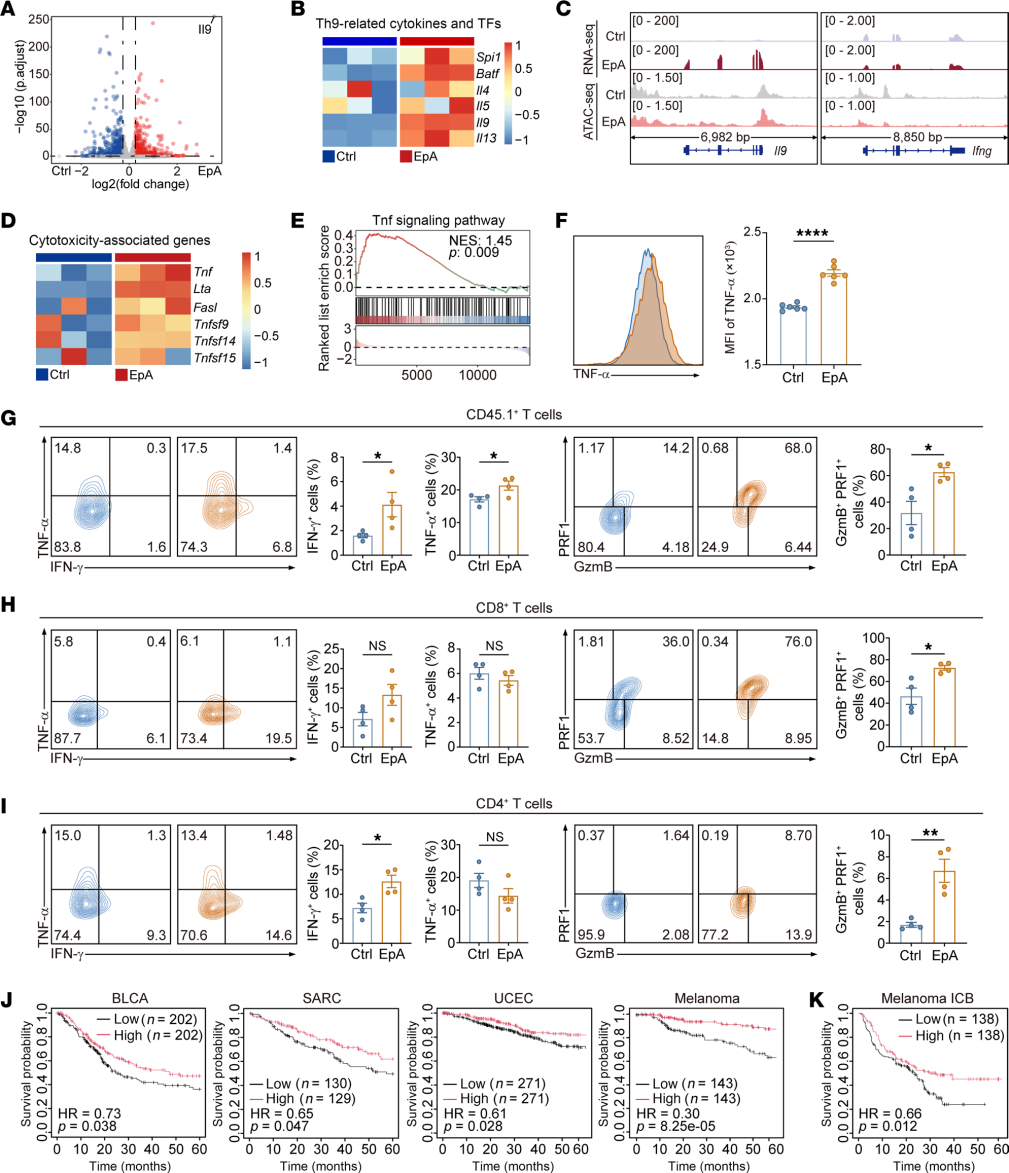
EpA promotes the differentiation of cytotoxic Th9 cells while reinforcing lineage identity. (**A**) Volcano plot depicting differentially expressed genes between EpA-treated Th9 cells and controls on day 4 in vitro polarization. (**B**) Heatmap showing normalized expression of Th9-related signature genes from RNA-seq analysis. (**C**) Integrated genome tracks illustrating transcript abundance and chromatin accessibility at the *Il9* and *Ifng* loci. (**D**) Heatmap of selected cytotoxicity-associated genes upregulated in EpA-treated Th9 cells. (**E**) GSEA revealing enrichment of the TNF signaling pathway in EpA-Th9 cells. (**F**) Representative flow cytometry and quantification of TNF-α expression in EpA-treated Th9 cells (mean ± SEM, *n* = 6). (**G**–**I**) Flow cytometric analysis of adoptively transferred CD45.1^+^ Th9 cells and endogenous T cells from spleens. EpA treatment increased IFN-γ^+^, TNF-α^+^, and granzyme B^+^ perforin^+^ populations among donor Th9 (**G**), endogenous CD8^+^ (**H**), and CD4^+^ T cells (**I**). Data in **G**–**I** are shown as mean ± SEM from *n* = 4 mice per group. (**J** and **K**) Kaplan-Meier survival analysis of patients with BLCA, SARC, and UCEC (**J**) and patients with melanoma and patients with melanoma receiving ICB therapy (**K**) based on the expression of canonical Th9-related signature genes (**B**) and cytotoxicity-associated genes (**D**) in EpA-treated Th9 cells. Statistical significance was determined using 2-tailed unpaired Student’s *t* test (**F**–**I**). *P < 0.05, **P < 0.01, ****P < 0.0001.

To elucidate the molecular mechanisms underlying the CD8^+^ T cell–dependent antitumor activity of EpA-Th9 cells, we performed integrated RNA-seq and transposase-accessible chromatin using sequencing (ATAC-seq) analyses to assess global transcriptional changes and chromatin accessibility. Principal component analysis revealed clear separation of gene expression between EpA-Th9 and control Th9 cells (Supplemental Figure 3A), consistent with widespread transcriptional divergence (Supplemental Figure 3B). Differential expression analysis identified 228 upregulated and 271 downregulated genes upon EpA treatment (Figure 3A). Notably, canonical Th9 cytokines — *Il9*, *Il4*, *Il5*, and *Il13* — as well as lineage-defining transcription factors, including *Spi1* and *Batf*, were upregulated (Figure 3B). GSEA confirmed activation of Th9-associated pathways, including enrichment of asthma-related signatures (Supplemental Figure 3C). Moreover, increased PD-1 expression indicated a more activated phenotype in EpA-Th9 cells (Supplemental Figure 3D).

ATAC-seq analysis revealed that global chromatin accessibility was only modestly affected by EpA (Supplemental Figure 3E). However, locus-specific inspection showed increased accessibility and transcription at the *Il9* locus, while both accessibility and expression of *Ifng* were further reduced — despite already being low in conventional Th9 cells — reinforcing Th9 identity (Figure 3C). Strikingly, EpA-Th9 cells exhibited strong upregulation of cytotoxic effector molecules, including *Tnf*, *Lta*, and *Fasl*, as well as activation of the TNF signaling pathway (Figure 3, D and E, and Supplemental Figure 3F). TNF-α induction was validated at the protein level (Figure 3F), indicating that EpA promotes a cytotoxic transcriptional program within the Th9 lineage, distinct from conventional Th1 conversion.

Given that Th1 cells are known to potentiate CD8^+^ CTL responses via IFN-γ and TNF-α, but Th9 cells generally lack this capability owing to limited Th1 transition in vivo, we hypothesized that EpA promotes deeper Th1-like differentiation in vivo. Supporting this, analysis of public transcriptomic datasets revealed that Th9 cells, despite displaying heightened TCR signaling signatures (Supplemental Figure 3G), expressed lower levels of cytotoxicity-associated genes and exhibited lower cytotoxic pathways compared with Th1 cells after transfer (Supplemental Figure 3, H–J) (15).

In contrast, adoptively transferred EpA-Th9 cells exhibited markedly increased expression of IFN-γ, TNF-α, and cytolytic molecules, including granzyme B and perforin, in the spleen compared with control Th9 cells (Figure 3G), indicative of enhanced Th1-like effector reprogramming. Within the tumor microenvironment, IFN-γ expression was also elevated in EpA-Th9 cells (Supplemental Figure 4A), whereas TNF-α levels remained unchanged (Supplemental Figure 4B). Notably, the frequency of granzyme B^+^ perforin^+^ EpA-Th9 cells was significantly higher in tumors (Supplemental Figure 4C), suggesting that EpA treatment promotes a cytotoxic effector phenotype.

To further examine whether EpA-Th9 cells influence endogenous T cell functionality, we analyzed the expression of IFN-γ, TNF-α, granzyme B, and perforin in host T cell populations. EpA-Th9 transfer led to a pronounced increase in granzyme B^+^ perforin^+^ CD8^+^ and CD4^+^ T cells in the spleen (Figure 3, H and I), with similar elevations observed within the tumor microenvironment (Supplemental Figure 4, D–I). In addition, both CD8^+^ and CD4^+^ T cells in tumors displayed upregulated TNF-α expression (Supplemental Figure 4E and H), further supporting an enhanced cytotoxic and proinflammatory response, although TNF-α expression in splenic T cells remained unchanged (Figure 3, H and I). Moreover, PD-1 expression was comparable between groups across both donor Th9 and endogenous T cell compartments (Supplemental Figure 4, J–L), indicating that the augmented effector activity induced by EpA occurred independently of changes in T cell exhaustion status.

To evaluate the clinical relevance of the EpA-induced cytotoxic Th9 program, we constructed a gene signature based on the upregulated genes in Figure 3, B and D, and applied it to publicly available datasets of patients with cancer. Patients stratified by high expression of this cytotoxic Th9 signature exhibited significantly improved overall survival across multiple cancer types, including bladder urothelial carcinoma (BLCA), sarcoma (SARC), uterine corpus endometrial carcinoma (UCEC), and melanoma (Figure 3J). Furthermore, patients with melanoma with elevated cytotoxic Th9 signature scores were predicted to derive greater clinical benefit from ICB therapy (Figure 3K). These analyses underscore the potential translational value of cytotoxic Th9 cells in human cancer immunotherapy.

Collectively, these results demonstrate that EpA drives the differentiation of a cytotoxic Th9 subset with enhanced Th1-like features and the capacity to license endogenous CD8^+^ T cell responses, thereby addressing a key limitation of conventional Th9 cells and offering a promising strategy for adoptive T cell therapy.

### EpA enhances the metabolic fitness of Th9 cells.

**Figure 4:**
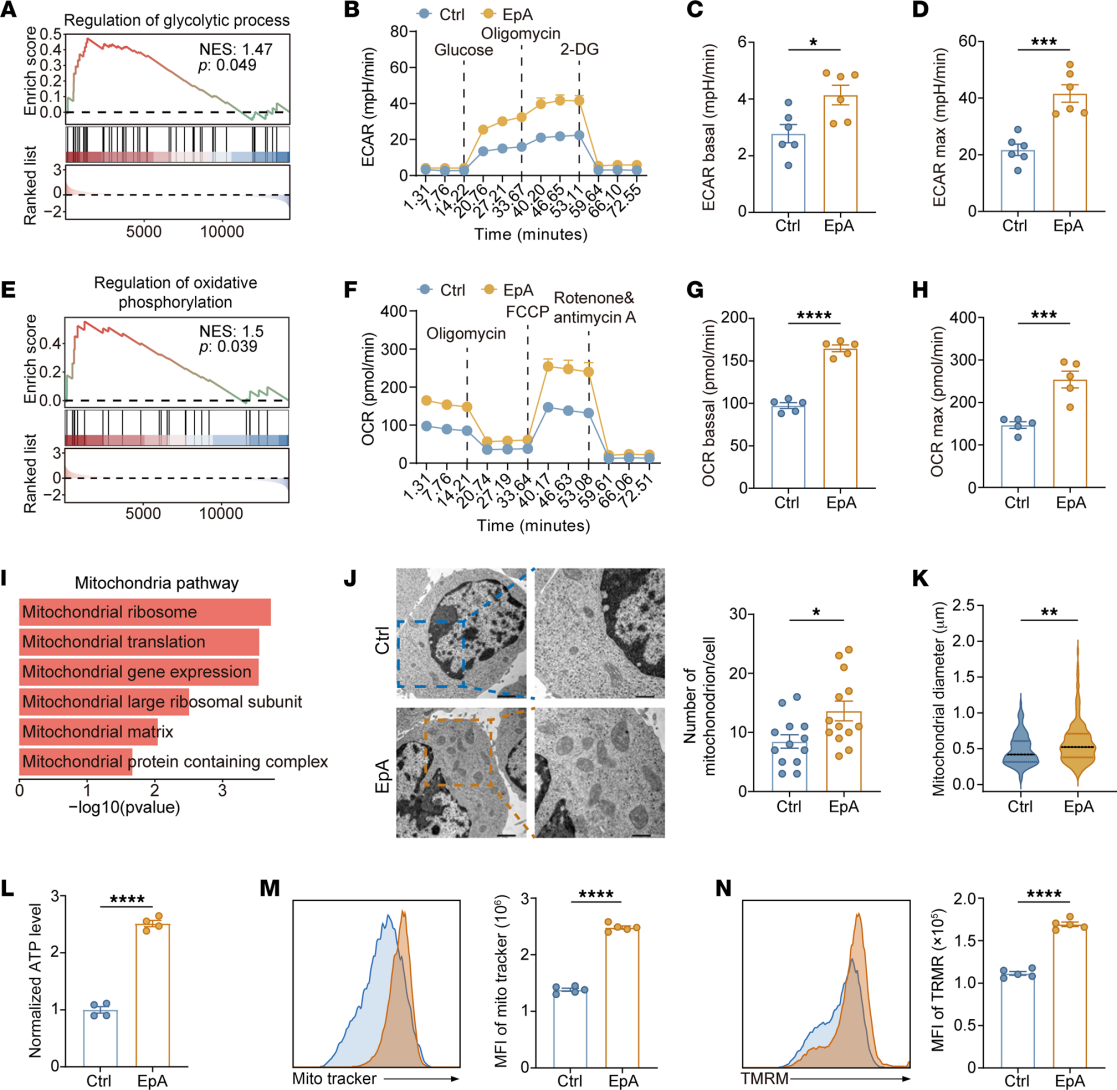
EpA enhances metabolic fitness in Th9 cells. (**A**) GSEA of RNA-seq data showing enrichment of glycolysis-related gene signatures in EpA-treated Th9 cells compared with controls. (**B**–**D**) Real-time extracellular acidification rate (ECAR) measurement using a Seahorse XF analyzer with sequential injection of glucose, oligomycin, and 2-deoxyglucose (2-DG) (**B**), followed by quantification of basal glycolysis (**C**) and glycolytic capacity (**D**) *(n* = 6). (**E**) GSEA of oxidative phosphorylation–associated gene sets in EpA-treated Th9 cells. (**F**–**H**) Oxygen consumption rate (OCR) analysis using a Seahorse XF analyzer with sequential addition of oligomycin, FCCP, and rotenone/antimycin A (**F**), followed by quantification of basal (**G**) and maximal respiration (**H**) (*n* = 5). (**I**) Pathway enrichment analysis of upregulated genes highlighting mitochondrial function–related categories. (**J** and **K**) Transmission electron microscopy (TEM) analysis of mitochondrial ultrastructure showing representative images and quantification of mitochondrial number per cell (**J**) and mitochondrial diameter (**K**). Scale bars: 1 μm (low magnification); 500 nm (high magnification) (*n* = 13 randomly selected microscope fields). (**L**) Intracellular ATP levels measured by a luminescence-based assay normalized to cell number (*n* = 4). (**M** and **N**) Flow-cytometric assessment of mitochondrial mass using MitoTracker Deep Red (**M**) and mitochondrial membrane potential using TMRM (**N**) (*n* = 5). Statistical significance was determined using 2-tailed unpaired Student’s *t* test (**C**, **D**, **G**, **H**, and **J**–**N**). Data are presented as mean ± SEM. *P < 0.05, **P < 0.01, ***P < 0.001, ****P < 0.0001.

T cell functionality is intimately linked to its metabolic state, which governs differentiation, persistence, and effector capacity (33–36). GSEA of transcriptomic data revealed significant enrichment of glycolytic and carbon metabolism pathways in EpA-treated Th9 cells (Figure 4A and Supplemental Figure 5A). This transcriptional shift was corroborated by extracellular flux analysis using the Seahorse platform, which demonstrated a marked increase in the extracellular acidification rate (ECAR) — a surrogate marker of glycolytic activity (Figure 4B). Both basal and maximal ECAR levels were significantly elevated in EpA-Th9 cells compared with controls (Figure 4, C and D), indicating enhanced glycolytic flux.

At the molecular level, SLC2A1 expression, which encodes the glucose transporter GLUT1 and is a critical regulator of glucose uptake, was markedly upregulated following EpA treatment (Supplemental Figure 5B), consistent with increased glucose metabolism.

Mitochondria serve as central hubs for bioenergetic control and are indispensable for sustaining T cell–mediated immune responses (37–41). Correspondingly, GSEA also highlighted upregulation of oxidative phosphorylation–related gene sets in EpA-Th9 cells (Figure 4E). Seahorse analysis of the oxygen consumption rate (OCR) further revealed higher basal and maximal respiration upon EpA treatment (Figure 4, F–H), reflecting enhanced mitochondrial oxidative capacity.

Moreover, pathway enrichment analysis identified multiple mitochondrial categories, including those involved in translation and ribosomal function (Figure 4I). Transmission electron microscopy directly confirmed an increased number and diameter of mitochondria in EpA-treated Th9 cells (Figure 4, J and K). Consistently, intracellular ATP levels were significantly elevated in EpA-Th9 cells (Figure 4L). Moreover, assessments of mitochondrial quality and functionality demonstrated elevated mitochondrial membrane potential and improved mitochondrial integrity in the EpA-Th9 group (Figure 4, M and N).

Collectively, these results demonstrate that EpA markedly enhances the metabolic fitness of Th9 cells by promoting both glycolytic and oxidative metabolism.

### EpA targets ZAP70 to amplify TCR-MEK-ERK signaling.

**Figure 5:**
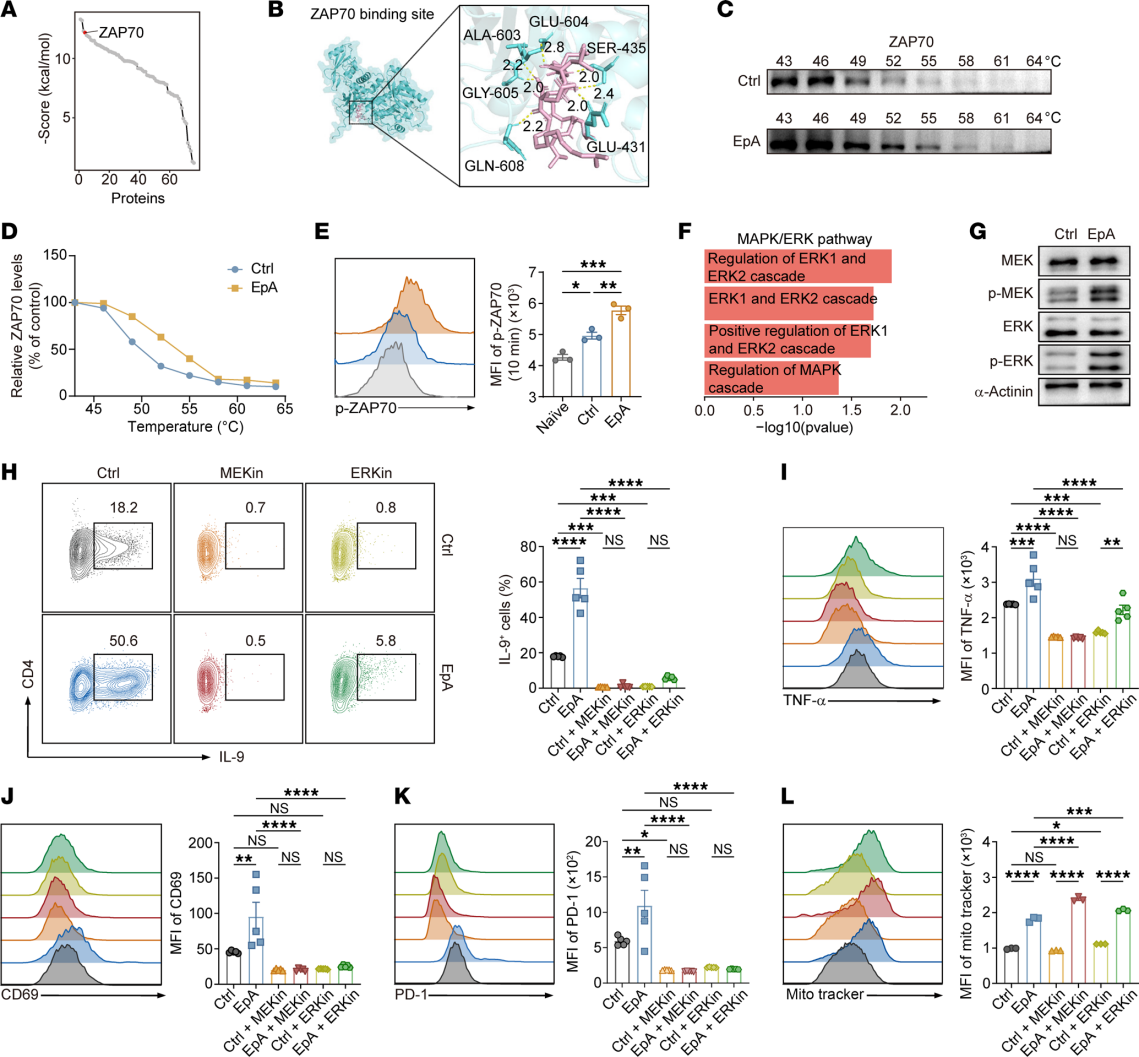
EpA promotes Th9 effector function by binding and activating ZAP70 in a MEK-ERK–dependent manner. (**A**) Identification and ranking of EpA-interacting proteins by affinity pulldown and LC-MS/MS, ordered by in silico binding energy predictions. The line graph shows proteins with binding energy scores of less than 0. (**B**) Predicted binding model of EpA (pink) within the kinase domain of murine ZAP70 (blue), with hydrogen bonding interactions shown as yellow dashed lines. (**C** and **D**) Cellular thermal shift assay (CETSA) of ZAP70 in Jurkat cell lysates treated with control or EpA, showing representative immunoblots (**C**) and quantification of normalized band intensities across the temperature gradient (**D**). (**E**) Flow-cytometric analysis of phosphorylated ZAP70 in Th9 cells 10 minutes after TCR activation (mean ± SEM, *n* = 3). (**F**) Pathway enrichment analysis of RNA-seq data showing MAPK/ERK-related pathways in EpA-treated Th9 cells. (**G**) Immunoblot analysis of total and phosphorylated MEK1/2 and ERK1/2 in control and EpA-treated Th9 cells. (**H**–**L**) Functional consequences of MEK or ERK inhibition in EpA-treated Th9 cells. Flow-cytometric quantification of IL-9 (**H**), TNF-α (**I**), CD69 (**J**), PD-1 (**K**), and mitochondrial membrane potential measured by MitoTracker (**L**) following treatment with EpA with or without MEK inhibitor (trametinib, 10 nM) or ERK inhibitor (temuterkib, 100 nM). Data in **H**–**K** are shown as mean ± SEM (*n* = 5) and in **L** as mean ± SEM (*n* = 3). Statistical significance was determined using 1-way ANOVA followed by Tukey’s test (**E**, **H**–**L**). *P < 0.05, **P < 0.01, ***P < 0.001, ****P < 0.0001.

To identify the molecular target of EpA in Th9 cells, we synthesized a biotin-conjugated EpA derivative and performed affinity purification using streptavidin-conjugated magnetic beads, followed by mass spectrometry analysis (Supplemental Figure 6 and Supplemental Figure 7A). Among the proteins specifically enriched by EpA pulldown, ZAP70 emerged as a top candidate based on both mass spectrometric abundance and high-affinity scores from molecular docking simulations (Figure 5A, Supplemental Figure 7B, and Supplemental Table 3), suggesting it as a primary target within the TCR signaling cascade (42).

In silico molecular docking confirmed strong binding between EpA and the kinase domain of ZAP70 (Figure 5B), and this interaction was experimentally validated by cellular thermal shift assay (CETSA), which showed increased thermal stability of ZAP70 in the presence of EpA, particularly in the 49°C–55°C range (Figure 5, C and D). These results strongly support direct binding of EpA to ZAP70.

To determine the functional consequence of this interaction, we evaluated ZAP70 phosphorylation in naive CD4^+^ T cells activated under Th9-polarizing conditions. EpA treatment significantly enhanced ZAP70 phosphorylation at 30 minutes after activation (Figure 5E), indicating potentiation of proximal TCR signaling. Transcriptomic data also revealed enrichment of calcium ion–binding gene sets, consistent with activated TCR downstream signaling (Supplemental Figure 7C) (42).

ZAP70 is known to phosphorylate LAT, initiating TCR signalosome formation and triggering downstream pathways such as NF-κB, PI3K/AKT, and MAPK/ERK (42). GSEA analysis indicated activation of the MAPK/ERK pathway (Figure 5F), which was confirmed by Western blot showing increased phosphorylation of MEK and ERK (Figure 5G) but not of AKT or NF-κB components (Supplemental Figure 7D). Given the key role of mTOR signaling downstream of TCR activation, we next examined mTOR activity. Both total and phosphorylated mTOR levels remained unchanged in EpA-treated Th9 cells (Supplemental Figure 7D), and GSEA similarly showed no enrichment of mTOR-related gene sets (Supplemental Figure 7E). Collectively, these results indicate that EpA specifically activates the MEK-ERK branch of TCR signaling without engaging the AKT, NF-κB, or mTOR pathways.

To assess the functional role of MEK-ERK signaling in EpA-Th9 polarization, we treated differentiating T cells with pharmacological inhibitors of MEK (trametinib) and ERK (temuterkib). Both inhibitors almost completely abrogated IL-9 production, and EpA was unable to restore it (Figure 5H), indicating that Th9 cell differentiation is MEK-ERK–dependent. Similarly, expression of TNF-α and activation markers CD69 and PD-1 were significantly reduced upon MEK/ERK blockade, even in the presence of EpA (Figure 5, I–K).

Interestingly, pharmacological inhibition of MEK/ERK signaling resulted in a paradoxical increase in mitochondrial membrane potential in EpA-treated Th9 cells (Figure 5L), indicating that this aspect of mitochondrial enhancement is independent of the ZAP70-MEK-ERK axis. While activation of this pathway promotes Th9 cell differentiation and effector function, it may concurrently impose constraints on mitochondrial biogenesis or activity. These findings suggest that EpA exerts its full immunometabolic effects through additional, yet unidentified, signaling mechanisms that cooperate with ZAP70-ERK activation to support Th9 cell metabolic fitness.

### SREBP1 is an additional functional target of EpA and regulates Th9 cell differentiation and mitochondrial fitness.

**Figure 6:**
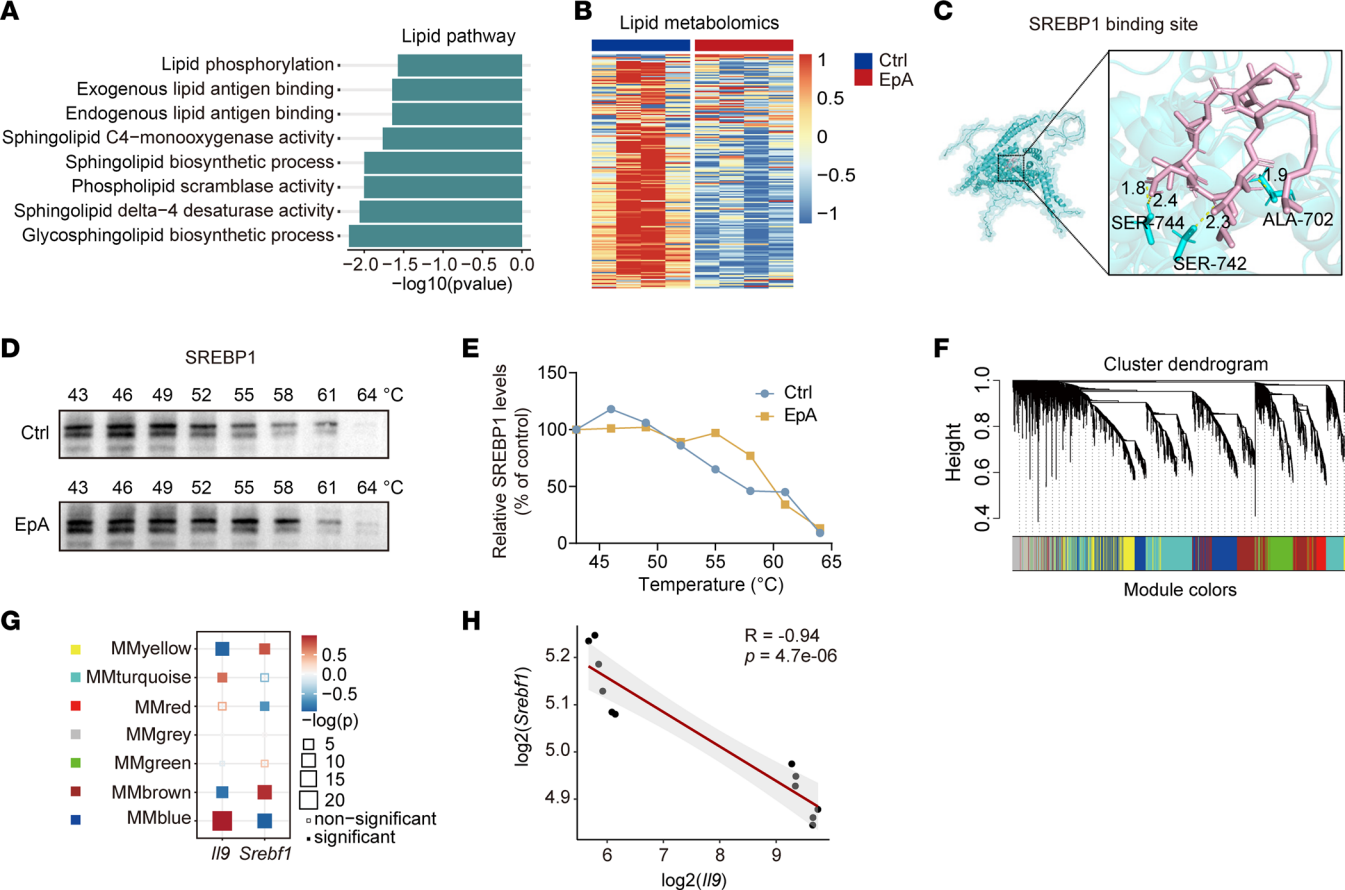
SREBP1 is an additional EpA target in Th9 cells. (**A**) Pathway analysis of RNA-seq data depicting of lipid metabolism pathways in EpA-treated Th9 cells. (**B**) Untargeted lipidomics profiling of Th9 cells on day 4 showing global differences in lipid species abundance between control and EpA-treated groups. (**C**) Predicted binding model of EpA (pink) within SREBP1 (blue). (**D** and **E**) CETSA validating EpA–SREBP1 binding. Representative Western blot (**D**) and quantification (**E**) of normalized SREBP1 signal intensities across a temperature gradient (°C). (**F**) Weighted gene coexpression network analysis (WGCNA) of RNA-seq datasets identifying 7 gene modules. (**G**) Module-trait correlation heatmap revealing a negative association between *Il9* and *Srebp1*-associated gene modules. (**H**) Validation of inverse correlation between *Il9* and *Srebf1* expression using RNA-seq data from 12 independent Th9 samples.

To uncover additional molecular targets of EpA, we examined RNA-seq data alongside relevant metabolic pathways. Notably, multiple lipid-related pathways were markedly downregulated after EpA treatment (Figure 6A). Downregulation trends were also observed for pathways related to long-chain fatty acid transport, intracellular sterol transport, and sterol transfer activity, though statistical significance was not reached (Supplemental Figure 8, A–C). Given recent reports that fatty acid biosynthesis restrains Th9 cell differentiation (43), we hypothesized that EpA enhances Th9 function, in part, by repressing lipid synthesis to improve metabolic fitness.

To explore this, we conducted untargeted lipidomic profiling of EpA-treated and control Th9 cells. EpA-Th9 cells exhibited a global reduction in lipid species, corroborating transcriptomic findings (Figure 6B). We next focused on transcription factors known to regulate lipid biosynthesis (44) — ChREBP, SREBP1, USF1, and LXR-α — and evaluated their potential interaction with EpA via in silico docking analysis (Figure 6C and Supplemental Figure 8, D–F). Among these, SREBP1 showed the second highest binding affinity but the most abundantly expressed, identifying it as the most likely regulatory target (Supplemental Table 4). This prediction was validated using CETSA, which revealed enhanced thermal stability of SREBP1 in the presence of EpA, indicating a direct binding interaction (Figure 6, D and E).

To assess the relationship between SREBP1 and Th9 cell differentiation, we performed weighted gene coexpression network analysis (WGCNA) of our RNA-seq data, which could be partitioned into 7 gene modules (Figure 6F). We analyzed the correlations between *Il9*, *Srebf1*, and the 7 modules and found that the modules positively correlated with *Il9* were negatively correlated with *Srebf1* (Figure 6G). The correlation analysis also suggested a potential negative correlation between *Il9* and *Srebf1* (Figure 6H).

**Figure 7:**
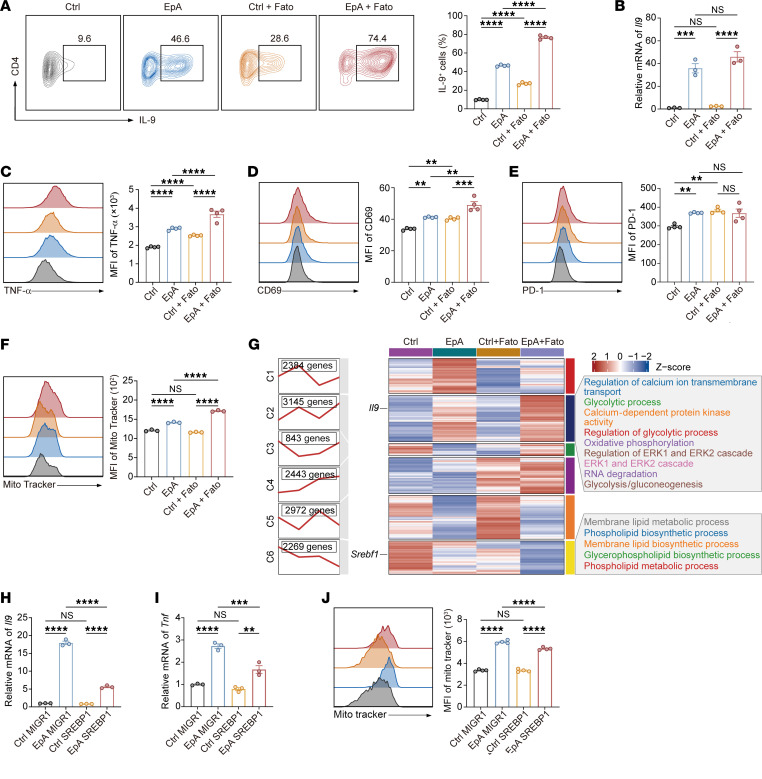
SREBP1 inhibition potentiates the immunometabolic effects of EpA in Th9 cells. (**A**) Flow-cytometric analysis of intracellular IL-9 expression in Th9 cells treated with vehicle, EpA (10 μM), fatostatin (5 μM), or both EpA and fatostatin, with representative plots and quantification (*n* = 4). (**B**) Quantitative PCR analysis of *Il9* mRNA expression in cells treated as in **A** (*n* = 3). (**C**–**F**) Flow cytometric analysis of TNF-α production (**C**), CD69 expression (**D**), PD-1 expression (**E**), and mitochondrial membrane potential measured by MitoTracker staining (**F**) in Th9 cells treated with the indicated conditions (*n* = 4 for **C**–**E**; *n* = 3 for **F**). (**G**) Hierarchical clustering heatmap of RNA-seq data from Th9 cells treated with vehicle, EpA, fatostatin, or both EpA and fatostatin. Cluster 2 includes genes related to calcium signaling, glycolysis, oxidative phosphorylation, and ERK signaling; cluster 6 includes genes associated with lipid biosynthesis. (**H**–**J**) *Il9* (**H**) and *Tnf* (**I**) mRNA expression and mitochondrial membrane potential (**J**) in Th9 cells overexpressing SREBP1, with or without EpA treatment (*n* = 3 for mRNA, *n* = 4 for mitochondrial analysis). Statistical significance was determined using 1-way ANOVA followed by Tukey’s post hoc test (**A**–**F** and **H**–**J**). Data are presented as mean ± SEM (**A**–**F** and **H**–**J**). **P < 0.01, ***P < 0.001, ****P < 0.0001.

To functionally validate SREBP1 as a mediator of EpA’s immunometabolic effects, we first employed fatostatin, a well-characterized pharmacological inhibitor of SREBP1. Inhibition of SREBP1 alone significantly upregulated IL-9 production in Th9 cells, and this effect was further enhanced when combined with EpA (Figure 7A). Consistently, *Il9* transcript levels showed a similar trend (Figure 7B). TNF-α production and CD69 expression were also elevated in the combination group, suggesting amplified effector activation (Figure 7, C and D). While PD-1 expression was not further increased in EpA-Th9 cells treated with fatostatin, fatostatin alone induced PD-1 upregulation in control Th9 cells (Figure 7E), indicating distinct regulatory effects depending on the activation context.

Strikingly, mitochondrial membrane potential was further augmented in EpA-Th9 cells upon SREBP1 inhibition (Figure 7F), indicating additive effects on mitochondrial fitness. This was accompanied by reduced expression of *Srebp1* as well as *Fads1*, a key enzyme in the synthesis of long-chain polyunsaturated fatty acids, reinforcing the inhibition of lipid anabolic programs (Supplemental Figure 8, G and H) (45). To assess the global transcriptional consequences of SREBP1 inhibition in combination with EpA, RNA-seq analysis was performed on 4 groups (vehicle control, EpA, fatostatin, and EpA and fatostatin). Gene clustering analysis revealed 6 distinct gene expression modules (Figure 7G). EpA treatment notably upregulated genes within cluster 2, which were further enhanced by fatostatin. This cluster was enriched for genes involved in calcium ion transport, glycolysis, oxidative phosphorylation, and ERK signaling — pathways critical for T cell effector function and metabolic activation. In contrast, genes involved in lipid biosynthesis were enriched in cluster, were progressively downregulated by EpA, and were further suppressed by fatostatin, indicating concerted repression of lipogenic programs.

Consistent with these findings, supplementation with oleic acid (OA), a downstream product of fatty acid biosynthesis (Supplemental Figure 8, I and J), reinforced the notion that lipid accumulation antagonizes Th9 polarization. Furthermore, SREBP1 overexpression markedly suppressed the EpA-induced increases in IL-9 and TNF-α at both the mRNA and protein levels (Figure 7, H and I, and Supplemental Figure 8, K–N) and abolished the EpA-induced enhancements in mitochondrial membrane potential (Figure 7J).

Together, these findings establish SREBP1 as a direct functional target of EpA. By repressing SREBP1 activity, EpA alleviates lipid-mediated metabolic burden, redirects biosynthetic flux toward oxidative metabolism, and stabilizes Th9 identity and mitochondrial fitness. This lipid-signaling axis works in concert with TCR-ZAP70-ERK activation to endow Th9 cells with a metabolically optimal, cytotoxic phenotype well-suited for adoptive immunotherapy.

### EpA promotes human Th9 cell differentiation and enhances tumor therapeutic efficacy.

**Figure 8:**
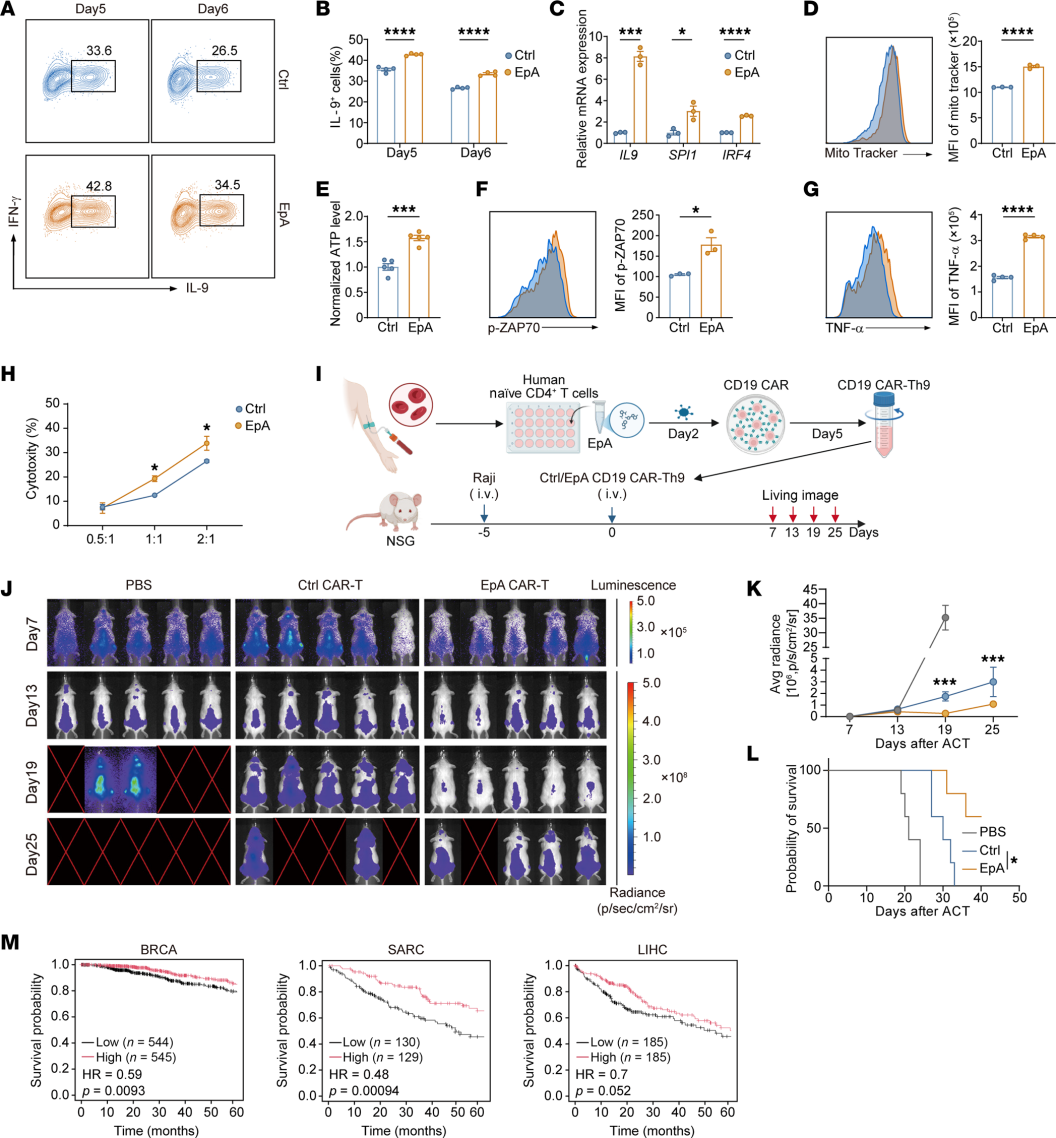
EpA promotes human Th9 cell differentiation and enhances the antitumor activity of Th9 CAR T cells. (**A** and **B**) Flow-cytometric analysis of IL-9 production in human CD4^+^ T cells cultured under Th9-polarizing conditions with or without EpA treatment during in vitro differentiation. Representative plots (**A**) and quantification (**B**) (*n* = 4). (**C**) Quantitative PCR analysis of *IL9*, *IRF4*, and *SPI1* mRNA expression in human Th9 cells (*n* = 3). (**D**) Flow-cytometric analysis of mitochondrial membrane potential in human Th9 cells (*n* = 3). (**E**) Luciferase-based assay analysis of intracellular ATP levels in human Th9 cells (*n* = 5). (**F**) Flow-cytometric analysis of phosphorylated ZAP70 in human Th9 cells (*n* = 3). (**G**) Flow-cytometric analysis of TNF-α expression in human Th9 cells (*n* = 4). (**H**) Cytotoxicity assay of EpA-treated or control CD19 CAR Th9 cells against Raji lymphoma target cells at indicated effector-to-target (E/T) ratios (*n* = 3). (**I**) Schematic diagram of the in vivo Raji-luciferase xenograft model in NSG mice receiving control or EpA-treated human Th9 CAR T cells. (**J** and **K**) Representative bioluminescence imaging (**J**) and quantification of tumor radiance (**K**) in NSG mice at indicated time points after adoptive transfer. (**L**) Kaplan-Meier survival curves of NSG mice treated as in **J**. Data in **J**–**L** are shown from *n* = 5 mice per group. (**M**) Kaplan-Meier survival analysis of patients with BRCA, SARC, and LIHC from the TCGA dataset based on the expression of the top 10 most upregulated genes (*Il9*, *H19*, *Fbxl21*, *Myh11*, *Lhx6*, *Rab27b*, *C1s1*, *Hao1*, *Ccdc159*, *Il1rn*) in EpA-Th9 cells. Statistical significance was determined using 2-tailed unpaired Student’s t test (**B**–**H**), 2-way ANOVA followed by Šidák’s post hoc test (**K**), and log-rank test (**L**). Data are presented as mean ± SEM. *P < 0.05, ***P < 0.001, ****P < 0.0001. Schematic in **I** created using BioRender (https://Biorender.com).

To assess the translational relevance of EpA, we evaluated its effects on human Th9 cell differentiation. Naive CD4^+^ T cells isolated from peripheral blood were cultured under Th9-polarizing conditions in the presence or absence of EpA. As previously reported, IL-9 production declined between day 5 and day 6 of culture (46); however, EpA-treated cells maintained significantly elevated IL-9 expression throughout this period (Figure 8, A and B). Correspondingly, EpA upregulated *IL9* and the Th9 lineage–defining transcription factors *IRF4* and *SPI1* at the mRNA level (Figure 8C). EpA also enhanced the metabolic profile of human Th9 cells, as shown by increased mitochondrial membrane potential, and ATP production (Figure 8, D and E), indicating improved metabolic fitness — an essential feature for sustained T cell function in adoptive immunotherapy. Similar to murine EpA-Th9 cells, human EpA-Th9 cells exhibited increased phosphorylation of ZAP70 and higher TNF-α expression (Figure 8, F and G). Furthermore, treatment with the SREBP1 inhibitor fatostatin further augmented IL-9 production and mitochondrial membrane potential in EpA-Th9 cells during polarization (Supplemental Figure 9, A and B). Collectively, these findings demonstrate that EpA promotes the generation of metabolically active and functionally competent Th9 cells in humans, consistent with its effects in mouse Th9 cells.

Given the clinical success of chimeric antigen receptor (CAR) T cell therapy, we next investigated whether EpA could enhance the performance of human Th9 CAR T cells. CD4^+^ Th9 cells were engineered to express a CD19-targeting CAR and treated with EpA during polarization (Supplemental Figure 9C). EpA-treated Th9 CAR T cells exhibited increased IL-9 production (Supplemental Figure 9, D and E), alongside elevated mitochondrial potential and ATP output (Supplemental Figure 9, F and G). Functionally, these cells demonstrated significantly greater cytotoxicity against Raji lymphoma cells in vitro (Figure 8H).

To evaluate antitumor activity in vivo, Raji-luciferase tumor cells were injected intravenously into NSG mice, followed by treatment with control or EpA-Th9 CAR T cells (Figure 8I). EpA-Th9 CAR T cell–treated mice showed markedly reduced tumor burden and significantly prolonged survival (Figure 8, J–L). Moreover, granzyme B levels were elevated in EpA-treated CAR T cells after transfer, indicating enhanced cytolytic function in vivo (Supplemental Figure 9, H and I). To further assess the clinical relevance of EpA-Th9–associated transcriptional changes, the top 10 most upregulated genes in EpA-Th9 cells were defined as constituting an EpA-Th9 gene signature. Kaplan-Meier survival analysis using The Cancer Genome Atlas (TCGA) dataset revealed that patients with higher expression of the EpA-Th9 signature exhibited improved overall survival in breast cancer (BRCA), liver hepatocellular carcinoma (LIHC), and SARC (Figure 8M), supporting the translational potential of EpA-enhanced Th9 cell therapy.

Collectively, these results demonstrate that EpA promotes the differentiation and metabolic activation of human Th9 cells and substantially improves the antitumor efficacy of Th9 CAR T cells both in vitro and in vivo.

## Discussion

Natural products remain a foundational source of immunomodulatory agents, and medicinal fungi such as *G*. *lucidum* have long been recognized for their immune-enhancing properties (17, 47). However, practical limitations, including slow cultivation, inconsistent compound yields, and complex extraction procedures, have hindered their clinical translation. In contrast, endophytic fungi that reside within medicinal plants or fungi offer a scalable and chemically diverse alternative, capable of producing structurally distinct bioactive metabolites under defined culture conditions (18, 27, 48, 49). While endophyte-derived cyclopeptides such as cyclosporine A have been historically utilized as immunosuppressants, their capacity to enhance T cell–mediated immunity has remained underexplored (50).

Here, we identify EpA, a cyclic peptide from *Trichoderma sp*. associated with *G*. *lucidum*, as a first-in-class immunoenhancing reagent that reprograms Th9 cells through dual modulation of TCR signaling and lipid metabolism. Th9 cells are metabolically resilient, stem-like CD4^+^ T cells with long-term persistence and superior tumor control, making them attractive candidates for ACT (12, 14–16). However, their low intrinsic cytotoxicity and limited ability to orchestrate CD8^+^ T cell responses pose key challenges. Previous studies have shown that Th9 cells function largely independently of CD8^+^ T cells in tumor models, a feature attributed to their type II cytokine dominance and partial Th1-like conversion in vivo (15). In contrast, EpA-Th9 cells acquired a distinct, cytotoxic phenotype, expressing IFN-γ, TNF-α, granzyme B, and perforin after adoptive transfer. Critically, EpA-Th9 cells mediate tumor regression in a CD8^+^ T cell–dependent manner, indicating a functional reorientation toward broader cytotoxic coordination. This CD8-licensing function is reminiscent of Th1 cells and suggests that EpA enhances Th9-mediated antitumor immunity not only through intrinsic effector programs, but also via extrinsic activation of CD8^+^ T cells through enhanced cytokine production, antigen presentation, and chemokine secretion.

At the mechanistic level, this reprogramming is driven by EpA’s dual targeting of ZAP70 and SREBP1, two critical regulators of T cell activation and metabolic adaptation. EpA promotes ZAP70 phosphorylation and activates the downstream MEK-ERK signaling axis, a pathway indispensable for Th9 polarization and IL-9 production. This is consistent with prior findings that the MEK-ERK cascade supports T cell activation, effector differentiation, and cytokine expression (51, 52). However, sustained ERK activation has also been implicated in T cell exhaustion and impaired mitochondrial integrity (52). Indeed, pharmacologic ERK inhibition in our system increased mitochondrial membrane potential, revealing an apparent contradiction.

Despite activating ERK signaling, EpA-treated Th9 cells retained superior mitochondrial mass, membrane potential, and ATP production, suggesting the involvement of a parallel pathway that supports mitochondrial fitness. Transcriptomic and biochemical analyzes revealed SREBP1, a master regulator of de novo lipogenesis, as a second direct target of EpA. SREBP1 plays a context-dependent role in T cell immunity: it is essential for the metabolic reprogramming of CD8^+^ T cells during activation, coordinating lipid biosynthesis with glycolytic and oxidative metabolism, though it does not directly affect mitochondrial mass in these cells (53). In Tregs, SREBP1 supports suppressive function within the tumor microenvironment by maintaining lipid-anabolic programs; however, SREBP1 deficiency in this context does not markedly alter mitochondrial mass, membrane potential, or reactive oxygen species production (54). Notably, SREBP1-driven lipogenesis consumes key metabolic intermediates, such as acetyl-CoA and NADPH, which are also essential substrates for mitochondrial oxidative phosphorylation (55). Moreover, lipid intermediates like malonyl-CoA inhibit fatty acid oxidation and promote endoplasmic reticulum stress, both of which can compromise mitochondrial function.

Although ZAP70-ERK signaling and SREBP1-mediated lipid synthesis were initially analyzed separately, our findings and previous reports indicate that they are mechanistically interconnected. Activation of the ZAP70-ERK pathway promotes transcription of Th9-defining factors such as IRF4 and BATF, thereby driving IL-9 expression and cytotoxic programming. However, excessive ERK activity can impose metabolic stress by upregulating SREBP1 and its downstream lipogenic genes (FASN, SCD) (56, 57), leading to lipid accumulation and mitochondrial dysfunction. Consistently, EpA-induced ERK activation alone reduced mitochondrial potential, whereas concurrent SREBP1 inhibition restored oxidative capacity and ATP production, suggesting a compensatory interplay between these pathways. Given that ERK phosphorylates and stabilizes SREBP1 (58–60), EpA likely fine-tunes ERK signaling to promote Th9 lineage commitment while concurrently restraining SREBP1 activity to preserve mitochondrial integrity. This coordinated signaling–metabolic crosstalk allows Th9 cells to maintain balanced activation and metabolic resilience, thereby sustaining durable cytotoxicity and enhancing cooperation with CD8^+^ T cells in tumor immunity. Collectively, these findings illustrate a broader immunometabolic principle: optimal T cell functionality arises not from dominance of a single pathway, but from the integrated tuning of signaling and metabolic networks.

The translational relevance of this dual-pathway strategy was validated in human T cells. EpA enhanced IL-9 expression, mitochondrial function, and Th9 polarization in primary human CD4^+^ T cells. Moreover, EpA markedly boosted the in vivo efficacy of human Th9 CAR T cells in a xenograft lymphoma model. These results demonstrate that EpA can serve not only as a Th9-differentiating agent, but also as a pharmacologic enhancer for CAR-based therapies.

In summary, we identify EpA as a valuable cyclic peptide that reprograms Th9 cells through cooperative modulation of TCR signaling and lipid metabolism. This dual mechanism supports both cytotoxic function and CD8^+^ T cell–dependent antitumor immunity, addressing key limitations of conventional Th9-based ACT. More broadly, our study illustrates how pharmacologic integration of immune signaling and metabolism can unlock latent potential within Th9 cells, offering a powerful strategy to enhance the efficacy of cellular immunotherapies.

## Methods

### Sex as a biological variable.

Human and mouse studies included both sexes; however, sex was not analyzed as a biological variable.

### Isolation and identification of endophytic fungi.

Detailed procedures for endophytic fungi isolation and identification, chemical experimental methods, extraction and isolation (including purification of EpA), and synthesis of the EpA-biotin molecular probe are described in Supplemental Methods.

### Mice and cell lines.

C57BL/6 mice were housed in specific pathogen–free conditions at the Laboratory Animal Center of Southern Medical University. M-NSG mice (catalog NM-NSG-001) were obtained from the Shanghai Model Organisms Center. OT-II transgenic mice [C57BL/6-Tg(TcraTcrb)425Cbn/J] and CD45.1 congenic mice (B6.SJL-Ptprca Pepcb/BoyJ) were provided by Bing Sun at the Chinese Academy of Sciences Center for Excellence in Molecular Cell Science, Shanghai, China, while β2m-KO (B6.129P2-B2mtm1Unc/DcrJ) mice were a gift from Mei Jiang at Shanghai General Hospital, Shanghai, China. All mice used in experiments were sex and age matched (6–8 weeks old).

B16 cells were purchased from ATCC, and Raji-CD19 cells were provided by Gang Xue at Suzhou Institute of Systems Medicine, Chinese Academy of Medical Sciences, Suzhou, China. B16-OVA cells were generated by viral transduction. B16, B16-OVA, and HEK293T cells were cultured in DMEM (ServiceBio, catalog G4511) supplemented with 10% heat-inactivated FBS (ExCell, catalog FSP500) and 100 U/mL penicillin-streptomycin (Gibco, Thermo Fisher Scientific, catalog 15140122). Raji-CD19 cells were maintained in RPMI-1640 medium (Gibco, catalog C11875500BT) under identical supplementation conditions.

### In vitro polarization of Th9, Th1, Th2, and Th17 cells and Tregs.

Naive CD4^+^ T cells were isolated from the spleens of C57BL/6J or CD45.1^+^ OT-II mice using the MojoSort Mouse CD4 Naive T Cell Isolation Kit (BioLegend, catalog 480040), following the manufacturer’s protocol. Purified cells were cultured in RPMI-1640 medium supplemented with 10% FBS, 1% penicillin-streptomycin, and 0.1% β-mercaptoethanol (Gibco, catalog 21985023).

T cell polarization was initiated under plate-bound anti-CD3 (2 μg/mL, clone 17A2, BioXCell, catalog BE0002) and soluble anti-CD28 (1 μg/mL, clone 37.51, BioXCell, catalog BE0015-1) stimulation, with the addition of cytokines and neutralizing antibodies specific to each subset. For Th9 cells, these included IL-4 (10 ng/mL, Peprotech, catalog 400-04), TGF-β (1 ng/mL, EPOTO Biotech, catalog HF-2021), anti–IL-12 (5 μg/mL, clone C17.8, BioXCell, catalog BE0051), and anti–IFN-γ (10 μg/mL, clone XMG1.2, BioXCell, catalog BE0055); for Th1 cells, these included IL-12 (10 ng/mL, GenScript, catalog Z03211-50) and anti–IL-4 (5 μg/mL, clone 11B11, BioXCell, catalog BE0045); for Th2 cells, these included IL-4 (50 ng/mL), anti–IFN-γ (20 μg /mL), anti–IL-12 (5 μg/mL), Th17 cells: IL-6 (20 ng/mL, EPOTO Biotech, catalog MF-1006), TGF-β (1 ng/mL), IL-1β (10 ng/mL, Peprotech, catalog AF-211-11B), anti–IFN-γ (10 μg/mL), anti–IL-12 (5 μg/mL), and anti–IL-4 (5 μg/mL); and for Tregs, these included TGF-β (3 ng/mL). After 3 days of initial culture, cells were either collected for downstream assays or continued in extended culture with refreshed cytokines to maintain lineage commitment.

During the entire culture period, experimental groups were treated continuously with the indicated compound at the designated concentration, whereas control groups received an equal volume of DMSO.

### Tumor inoculation and therapy.

C57BL/6 mice were subcutaneously injected with 5 × 10^5^ B16-OVA melanoma cells. Five days after tumor implantation, mice received an intravenous infusion of 2 × 10^6^ in vitro–differentiated Th9 cells (cultured for 5 days), which were derived from OT-II CD45.1 donor mice, accompanied by 4 × 10^5^ OVA peptide–pulsed DCs via the tail vein. To enhance T cell engraftment and facilitate lymphodepletion, a single intraperitoneal injection of cyclophosphamide (200 mg/kg, Sigma, catalog C0768) was administered 1 day prior to T cell transfer. Mice were euthanized at designated time points for the collection and analysis of spleens, tumor-draining lymph nodes, and tumor tissues.

For xenograft models, M-NSG mice were intravenously inoculated with 1 × 10^6^ Raji-CD19 tumor cells. On day 5 after inoculation, 2 × 10^6^ CD19-CAR Th9 cells were administered intravenously to evaluate in vivo therapeutic efficacy. Tumor progression and immune cell infiltration were subsequently monitored according to experimental endpoints.

### Bulk RNA-seq and transcriptomic analysis.

Total RNA was isolated from in vitro–differentiated Th9 cells treated with DMSO (control), EpA, fatostatin, or the EpA-fatostatin combination on day 4 of culture using RNAex Pro Reagent (AG, catalog AG21102), according to the manufacturer’s instructions. RNA integrity and concentration were assessed by Majorbio Technologies. RNA-seq libraries were constructed following standard Illumina protocols and sequenced using a paired-end strategy on an Illumina platform.

Raw sequencing reads were quality filtered and adapter trimmed using Trim Galore (v0.6.x) and then aligned to the mouse reference genome (GRCm38) with HISAT2 (v2.2.x). Gene-level quantification was performed using featureCounts (v2.0.x) to obtain raw read counts, which were subsequently normalized as fragments per kilobase of transcript per million mapped reads for exploratory analyses and data visualization.

Differential gene expression analysis was conducted using DESeq2 (v1.44.0) in R, with significance thresholds set at an adjusted *P* < 0.05 and absolute fold change ≥1.5. Visualization of transcriptomic profiles included volcano plots generated via ggplot2 (v3.5.1) and heatmaps rendered using pheatmap (v1.0.12), based on raw expression values.

### Functional enrichment analysis.

GSEA was performed using the clusterProfiler R package (v4.12.6), with curated gene sets obtained from the Gene Ontology and Kyoto Encyclopedia of Genes and Genomes databases. Enrichment plots were generated using the GseaVis R package (v0.1.0) to facilitate data visualization.

### Gene expression correlation analysis.

Gene expression values were log_2_ transformed to normalize distribution and stabilize variance across samples. The Pearson correlation coefficient was used to assess relationships between gene expression variables. Regression plots were generated using the ggplot2 R package (v3.5.1).

### WGCNA analysis.

WGCNA was conducted using the R package WGCNA (v1.72.1) to identify gene modules associated with phenotypic traits. Genes exhibiting the highest variance across samples were selected for network construction. An unsigned coexpression network was built by applying a soft-thresholding power to approximate scale-free topology. Modules were defined via hierarchical clustering with dynamic tree cut, and subsequent module-trait correlation analysis was performed to identify modules significantly associated with genes.

### Gene clustering analysis.

Gene expression clustering was conducted using the R package ClusterGVis (v0.1.2).

### Kaplan-Meier survival curve analysis.

For BLCA, SARC, UCEC, BRCA, LIHC, and melanoma ICB cohorts, survival curves were analyzed using the website Kaplan-Meier Plotter (61). Data related to patients with melanoma (not available on the website Kaplan-Meier Plotter) were downloaded from TCGA database using the TCGAbiolinks (v2.32.0) R package and analyzed with the survminer (v0.4.9) and survival (v3.6) R packages. Patients were stratified into high- and low-expression groups based on median gene expression values. Five-year overall survival was assessed for all cohorts.

### Bulk ATAC-seq.

Raw FASTQ reads were subjected to quality control using Trim Galore to remove adapter sequences and low-quality bases. Cleaned reads were aligned to the GRCm38 mouse reference genome using Bowtie2. Low-quality alignments were filtered out using SAMtools, and PCR duplicates were removed using Sambamba to reduce technical artifacts. ENCODE blacklist regions were excluded using BEDtools. Read positions were adjusted using alignmentSieve, followed by sorting and indexing with SAMtools to generate BAM files. Peak calling was conducted using MACS3 to identify regions of open chromatin indicative of accessible regulatory elements.

### Lipid metabolomics.

Comprehensive lipidomic profiling was performed using a liquid chromatography–mass spectrometry (LC-MS) platform. Briefly, samples were first subjected to protein precipitation and impurity removal to ensure metabolite integrity. Lipid species were then extracted using a solvent-based protocol optimized for broad lipid class coverage. LC-MS analysis was conducted in both positive and negative ionization modes to maximize lipid detection breadth. Mass spectrometry data, including MS and MS/MS spectra, were acquired using a high-resolution instrument. Lipid identification and quantification were performed using LipidSearch software (Thermo Fisher Scientific), which generated a lipid expression matrix for downstream statistical and pathway analysis.

### Viral production and transduction.

For murine T cell transduction, MIGR1-based retroviral vector encoding Srebf1 was used. HEK293T cells were transfected with these constructs using Lipofectamine 3000 (Thermo Fisher Scientific) to produce viral particles. Supernatants were collected between 48 and 72 hours after transfection, filtered through a 0.45 μm membrane, and stored at –80°C. Naive murine CD4^+^ T cells were activated with plate-bound anti-CD3 (2 μg/mL, clone 17A2, BioXCell) and soluble anti-CD28 (1 μg/mL, clone 37.51, BioXCell) stimulation in the presence of subset-specific polarizing cytokines and then transduced with viral supernatant supplemented with polybrene (10 μg/mL).

To generate human CAR T cells, a CD19-specific CAR construct — comprising a single-chain variable fragment targeting CD19, the costimulatory domain 4-1BB, and the CD3ζ signaling domain — was cloned into the lentiviral vector pUltra. Lentiviral particles were produced by transient cotransfection of HEK293T cells seeded in 6-well plates using X-tremeGENE HP DNA Transfection Reagent (Roche), according to the manufacturer’s protocol. Viral supernatants were collected 48 hours after transfection, filtered, and stored at –80°C until use.

For infection, human T cells were activated for 40 hours with plate-bound anti-CD3 (BioLegend, clone OKT3, 5 μg/mL) and soluble anti-CD28 antibodies (BioLegend, clone CD28.2, 2 μg/mL) antibodies and then resuspended in viral supernatant (MOI = 5) supplemented with recombinant human IL-2 (100 U/mL) and polybrene (10 μg/mL). The cell-virus mixture was subjected to spinoculation at 550*g* for 2 hours at room temperature, followed by incubation for 24 hours. Transduced CAR T cells were harvested on days 5 or 7 after activation for downstream functional assays.

### Human naive CD4^+^ T cell isolation and Th9 polarization.

Naive human CD4^+^ T cells were isolated from PBMCs obtained from healthy donors using the EasySep Human Naive CD4^+^ T Cell Isolation Kit II (STEMCELL Technologies, catalog 17555), according to the manufacturer’s protocol. Isolated T cells were cultured in OptiVitro T Cell Medium SF (Excell Bio, catalog TE000-N022) supplemented with recombinant human IL-2 (100 U/mL, Novoprotein, catalog C013). T cell activation was achieved using plate-bound anti-CD3 (5 μg/mL) and soluble anti-CD28 antibodies (2 μg/mL).

For Th9 polarization, cultures were supplemented with IL-4 (10 ng/mL, PeproTech, catalog AF-200-04), TGF-β (1 ng/mL), and neutralizing anti–IFN-γ monoclonal antibody (10 μg/mL, clone B27, BioXcell, catalog BE0245). After 3 days of initial culture, cells were either collected for downstream assays or continued in extended culture with refreshed cytokines to maintain lineage commitment without antibodies. Th9 cells were collected at days 4, 5, and 6 for subsequent in vitro assays. For adoptive transfer studies, day 4–cultured Th9 cells were washed and prepared for intravenous injection.

### Flow cytometry.

Details regarding staining panels (human and mouse), reagents (including clones and catalog numbers), acquisition settings, compensation/controls, and analysis procedures are provided in Supplemental Methods.

### Seahorse extracellular flux analysis.

CD4^+^ T cells were pre-activated for 4 days with plate-bound anti-CD3 and soluble anti-CD28 in the presence of DMSO or 10 μM EpA. After stimulation, cells were washed, resuspended in Seahorse XF assay medium (Agilent Technologies; pH 7.40–7.45, catalog 103576-100) and seeded at 5 × 10^5^ cells per well in Seahorse XF96 microplates. Plates were centrifuged at 400 × g for 5 minutes to promote adherence and monolayer formation, and all conditions were tested in quadruplicate.

Glycolytic activity was measured on a Seahorse XFe96 Extracellular Flux Analyzer (Agilent Technologies) using the XFe96 Glycolysis Stress Test kit (Agilent Technologies, catalog 103020-100) per the manufacturer’s instructions. Sequential injections of glucose (10 mM), oligomycin (1 μM), and 2-deoxyglucose (2-DG; 50 mM) were administered to determine ECAR as a proxy for glycolytic function. A glycolytic index was calculated from ECAR values obtained before and after each injection.

Oxidative phosphorylation was assessed on the same instrument using the XFe96 Mitochondrial Stress Test Kit (Agilent Technologies, catalog 103015-100). Oligomycin (1.5 μM), FCCP (0.5 μM), and rotenone plus antimycin A (0.5 μM each) were injected sequentially to measure OCR as a proxy for oxidative phosphorylation. The mitochondrial stress response was derived from OCR values obtained before and after each injection.

### RNA extraction and quantitative PCR.

Total RNA was extracted from T cells using RNAex Pro Reagent (AG, catalog AG21102) following the manufacturer’s instructions. First-strand complementary DNA (cDNA) was synthesized using the Hifair III 1st Strand cDNA Synthesis SuperMix (YEASEN, catalog 11137ES10). Quantitative real-time PCR (qRT-PCR) was conducted on a QuantStudio 3 Real-Time PCR System (Thermo Fisher Scientific) using ChamQ Universal SYBR qPCR Master Mix (Vazyme, catalog Q711-02) and gene-specific primers (Tsingke Biotech).

For normalization, mouse samples were referenced to *Gapdh* or *Actb*, and human samples were normalized to *GAPDH*. Relative gene expression was calculated using the ΔΔCt method. Primer sequences are listed in Supplemental Table 5.

### Western blotting.

Detailed lysis conditions, SDS–PAGE and transfer procedures, primary/secondary antibodies (including clones and catalog numbers), molecular weight markers, ECL detection, and ImageJ (NIH) quantification are described in Supplemental Methods.

### CETSA.

Jurkat or 293T cells were washed twice with PBS and resuspended in PBS containing protease and phosphatase inhibitors. Cells were lysed by 3 cycles of liquid nitrogen snap-freezing and thawing at room temperature. After centrifugation to remove debris, supernatants were divided into aliquots, treated with DMSO or EpA, and rotated at room temperature for 1 hour. One aliquot from each group was maintained at room temperature, and the others were heated for 3 minutes at 3 °C increments from 43°C to 64°C. Samples were then analyzed by SDS-PAGE and immunoblotting to assess protein thermal stability and compound binding.

### ATP assay.

Intracellular adenosine triphosphate (ATP) levels were measured using a chemiluminescent ATP assay kit (Beyotime, catalog S0026) according to the manufacturer’s protocol. Briefly, cells were lysed and incubated with the luciferase-based detection reagent, and the resulting luminescent signal was detected using an Infinite M200 multimode microplate reader (TECAN). ATP concentrations were calculated based on a standard curve and normalized to cell number or protein content where applicable.

### Mitochondrial measurement.

Mitochondrial mass and membrane potential were assessed using MitoTracker™ Deep Red FM (Thermo Fisher Scientific, catalog M22426) and TMRM (tetramethylrhodamine methyl ester; Macklin, catalog T868125), respectively, following the manufacturers’ protocols. Cells were incubated with the respective dyes, washed, and subjected to flow cytometry or fluorescence microscopy analysis. For ultrastructural assessment, mitochondrial number and diameter were quantified from high-resolution transmission electron microscopy (TEM) images using ImageJ software.

### Identification of EpA-binding proteins by pulldown and LC-MS/MS.

Cell lysates from Th9 cells were incubated overnight at 4 °C with EpA-biotin or biotin alone (control). Streptavidin-conjugated magnetic beads were added to capture biotinylated compounds and their interacting proteins. After washing, the bead-bound proteins were resolved by SDS-PAGE and visualized by silver staining. Distinct protein bands were excised, subjected to in-gel enzymatic digestion, and analyzed via liquid chromatography–tandem mass spectrometry (LC-MS/MS) to identify putative EpA-binding proteins.

### Molecular docking analysis.

The 3D structure of SREBP1 was predicted using AlphaFold v2.3.2, along with selected EpA-binding proteins identified by EpA-biotin pulldown assays. Protein structures were processed in PyMOL to remove water molecules and nonessential ligands, followed by the addition of polar hydrogens and Kollman charges. Energy minimization was performed in AutoDock Tools (Python Molecular Viewer), and the structures were saved in PDBQT format.

The 2D structure of EpA was obtained from the PubChem database (https://pubchem.ncbi.nlm.nih.gov/compound/129908858), converted to PDB format in PyMOL, protonated at pH 7, assigned torsional flexibility, and energy-minimized in AutoDock Tools before conversion to PDBQT.

Binding pockets were predicted using the Grid-based HECOMi Finder, and pocket coordinates were visualized in PyMOL to define docking grid boxes. Molecular docking was performed with AutoDock 4.2.6 using the Lamarckian Genetic Algorithm and default parameters, treating proteins as rigid and the ligand as fully flexible. For each target, 100 docking runs were conducted, and the lowest-energy poses were selected for further analysis. EpA-binding sites on pulldown-identified proteins, including ZAP70, were visualized in PyMOL (v2.5 or later).

### Fatty acid treatment.

OA (Sigma-Aldrich, catalog O1008) was first dissolved in 0.1 M NaOH and subsequently complexed with 20% fatty acid–free BSA to generate a bioavailable OA-BSA solution. OA was added to cell cultures at a final concentration of 50 μM, 24 hours after initial T cell activation.

### Luciferase-based cytotoxicity assay.

The cytotoxic activity of CD19-targeting CAR T cells was evaluated using a luciferase-based bioluminescence assay. Raji cells stably expressing firefly luciferase (ffLuc) were resuspended in RPMI 1640 medium supplemented with 10% FBS and seeded into 24-well plates. CAR T cells were added at various effector-to-target (E:T) ratios, while tumor-only wells were included as negative controls. After 20 hours of coculture, 20,000 cells from each tumor-only control well were harvested, washed with PBS, resuspended in 100 μL of lysis buffer, and transferred to a black 96-well plate. Luminescence intensity was measured using a multimode plate reader, and the percentage of specific lysis was calculated as follows: % Cytotoxicity = 1 – (luminescence of coculture well/luminescence of tumor-only control well).

### Serum collection and IL-6 quantification by ELISA.

Peripheral blood was collected from mouse tail veins at predefined time points in the tumor model. Samples were allowed to clot at 4°C for 1 hour and were then centrifuged at 600*g* for 25 minutes to obtain serum. Serum IL-6 levels were measured using an ELISA kit (Invitrogen; catalog 88-7064) according to the manufacturer’s instructions.

### Additional reagents and inhibitors.

The following pharmacological inhibitors were used at the indicated final concentrations: trametinib (MEK1/2 inhibitor; Selleck, catalog S4484) at 10 nM; temuterkib (ERK1/2 inhibitor; MCE, catalog HY-101494) at 100 nM; and fatostatin hydrobromide (SREBP inhibitor; MCE, catalog HY-14452A) at 5 μM. Dead cells were detected using Fixable Viability Dye eFluor 780 (eBioscience, catalog 65-0865).

### Statistics.

Statistical analysis was performed using GraphPad Prism 9, with 2-tailed Student’s *t* test, 1-way ANOVA, 2-way ANOVA, and log-rank test as appropriate. A *P* value less than 0.05 was considered statistically significant.

### Study approval.

All studies involving human samples were approved by the Ethics Committee of Nanfang Hospital, Southern Medical University (approval no. NFEC-2025-131) and conducted in accordance with institutional and international ethical guidelines. Animal experiments were approved by the Institutional Animal Care and Use Committee at the Laboratory Animal Center of Southern Medical University (approval no. SMUL202406037) and were performed in accordance with institutional guidelines and the approved experimental animal research protocol.

### Data availability.

The bulk RNA-seq and ATAC-seq datasets generated in this study have been deposited in the Gene Expression Omnibus (GEO) under accession numbers GSE295054 and GSE296515. Publicly available RNA-seq data for Th1 and Th9 cells can be accessed under GEO accession number GSE97087 (15). The values for all data points in the graphs are reported in the Supporting Data Values file.

## Author contributions

EB, QL, and TO initiated the study, designed the experiments, and wrote the paper. WZ, YC, and YS performed most of the experiments and statistical analyses. Yang Zhou performed all the bioinformatic analysis. JT, Yihan Zhu, JR, TD, HW, and YG helped with the experiments. YH and LJ provided critical suggestions.

## Funding support

- National Natural Science Foundation of China (82372775, 82172709).
- Guangdong Basic and Applied Basic Research Foundation (2023A1515012514).
- Guangzhou Key Research and Development Science Foundation (2023B1515120053).
- Guangdong Medical Scientific Research Foundation (A2023131).

## Supplementary Material

Supplemental data

Unedited blot and gel images

Supporting data values

## Version 1 12/09/2025

In-Press Preview

## Version 2 02/02/2026

Electronic publication
