# Supplementary material for: A fungi-derived cyclic peptide enhances Th9-mediated antitumor immunity by targeting ZAP70 and SREBP1
Source: J Clin Invest. 2025 Dec 9;136(3):e196907. doi: 10.1172/JCI196907 (PMC12867150; doi:10.1172/JCI196907)
Supplement: Supplemental data [file jci-136-196907-s131.pdf]

Supplemental Information

A fungal-derived cyclic peptide enhances Th9-mediated antitumor immunity by targeting ZAP70 and SREBP1

Wenli Zhao, Yang Zhou, Yuyang Chen, Yicheng Sun, Jiaxin Tang, Yihan Zhu, Jie Ren, Tianxu Du, Handuo Wang, Yuan Gao, Yu Hu, Ling Jiang, Tomohiko Ohwada, Qi Luo, Enguang Bi

Supplemental Methods

Isolation and Identification of Endophytic Fungi

The fresh fruiting bodies of *G. lucidum* were collected from the Lingzhi Planting Base of Tianren, Baiyun District, Guangzhou, China, in July 2021, and *Periplaneta americana* captured in the Southern Medical University, Guangdong Province, People's Republic of China, in November 2020*,* which was identified by ITS sequence analysis. The strain was deposited at Guangdong Provincial Key Laboratory of Chinese Medicine Pharmaceutics*, Southern Medical University*. Surface disinfection was performed using 70% ethanol for 1 min and then in 2.5% sodium hypochlorite solution for 5 min. Under the aseptic condition, the disinfected fruiting bodies or intestinal section were cut into small pieces and inoculated onto potato dextrose agar (PDA) plates. The inoculated medium was cultured at 28 °C for 2-7 days. Then, colonies were selected and subjected to purification procedures, resulting in the isolation of endophytic fungi, such as *Endomelanconiopsis* sp., *Trichoderma sp.*, *Talaromyces sp.*, *Nigrospora oryzae*, and *Mucor irregularis*.

Chemical Experimental Procedures

Column chromatography (CC) was performed on MCI gel CHP 20P (75-150 μm, Tokyo, Japan), RP−18 (50 mm, YMC, Japan), Sephadex LH-20 (Amersham Biosciences, Sweden), and silica gel (200–300 mesh). Semipreparative HPLC was carried out using a Shimadzu chromatograph with a 250 mm × 10 mm, i.d., 5 μm, YMC-Pak ODS-A, with a flow rate of 2.5 mL/min. NMR spectra were recorded on a Buker Avance 400 or 600 MHz instruments, with Tetramethylsilane (TMS) as an internal standard, and chemical shifts (δ) were expressed in ppm. All the spectra were analyzed by MestReNova 14.0.

Extraction

The endophytic fungi were cultured on potato dextrose agar (PDA) plate at 28 ^{o}C for 4 days. Subsequent the mycelia were added to 4 × 500 mL Erlenmeyer flasks each

containing 100 mL potato dextrose (PD) medium (2% glucose, 20% potato), and cultures were fermented by using a thermostatic shaker (200 r/min) for 3 days at 28 °C. And then 3 mL of mycelia suspension was transferred into 200 × 1 L Erlenmeyer flasks each containing rice medium (rice 60.0 g, glucose 0.3 g, yeast extract 0.3 g, H_2O 80.0 mL) for 30 days at 28 °C. Next, the rice medium was soaked for five times with methanol (5 × 30 L) at room temperature to provide crude extract, which was suspended in H_2O and extracted with ethyl acetate (three times) to obtain the crude extract. The extract was eluted by column chromatography on MCI gel CHP 20P with MeOH/H_2O gradient system (20:80–100:0) to obtain subfractions. Subsequently, they were further purified through other chromatography columns (Sephadex LH-20 and silica gel) and high-performance liquid chromatography (HPLC). Among them, endomelipeptide A (8.0 mg) was obtained by semi-preparative HPLC on YMC-Pack ODS-A (MeOH/H_2O containing 0.1% formic acid, 91%; t_R = 30.0 min, flow rate: 2.5 mL/min).

Synthesis of molecular probe

EpA (**1**) (2.1 mg, 1.0 eq) was dissolved in 1 mL of anhydrous *N*,*N*-dimethylformamide (DMF). 2-(7-azabenzotriazol-1-yl)-*N,N,N',N'*-tetramethyluronium hexafluorophosphate (HATU) (11.4 mg, 1.5 eq), triethylamine (TEA, 14.5 μL), and 4-dimethylaminopyridine (DMAP, 3.6 mg, 1.5 eq) were added sequentially. The mixture was stirred at room temperature for 30 minutes, followed by the addition of Biotin-*N*-PEG2-OH (9.9 mg, 1.5 eq). The reaction was allowed to proceed for 8 hours. After completion, the reaction mixture was poured into ice-water and extracted three times with ethyl acetate. The organic phases were washed three times with saturated brine to remove residual DMF, then dried over anhydrous magnesium sulfate. Purification by silica gel column chromatography (PE:EA = 20:1) afforded EpA-biotin (0.7 mg, a white solid).

Flow Cytometry

Cells were centrifuged, washed with FACS buffer (PBS containing 2% FBS and 2 mM EDTA), and incubated with Fc-blocking reagents (Human TruStain FcX™,

BioLegend, Cat. No. 422301 or Mouse TruStain FcX™, BioLegend, Cat. No. 101319) for 10 minutes at 4 ° C. For surface marker detection, cells were stained with fluorochrome-conjugated monoclonal antibodies in 96-well V-bottom plates and incubated on ice for 30 minutes. After staining, cells were washed twice and resuspended in FACS buffer for analysis.

Prior to intracellular cytokine staining, cells were restimulated for 4 hours with PMA (Absin, Cat. No. abs9107), ionomycin (Sigma-Aldrich, Cat. No. I0634), and brefeldin A (MedChemExpress, Cat. No. HY-16592). For intracellular cytokine staining, cells were first surface-stained as described above, then fixed and permeabilized using the BD Cytofix/Cytoperm™ kit (BD Biosciences, Cat. No. 554714) according to the manufacturer's protocol. Intracellular proteins were stained with directly conjugated antibodies targeting IL-9, IL-17a, IL-4, IFN-γ, TNF-α, FOXP3, p-ZAP70, Perforin, and Granzyme B.

Antibodies used for mouse samples included those against IL-9 (PE, clone RM9A4, Biolegend, Cat. No. 514104), IFN-γ (PE, clone XMG1.2, Biolegend, Cat. No. 505808), IL-4 (PE, clone 11B11, Biolegend, Cat. No. 504103), IL-17a (PE, clone TC-18H10.1, Biolegend, Cat. No. 506904), CD45.1 (BV421, clone A20, Biolegend, Cat. No. 110732; PE, clone A20, Biolegend, Cat. No. 110708; BV450, clone E-AB-F1184UQ, Elabscience, Cat. No. AF19855;), CD45 (BV510, clone 30-F11, Biolegend, Cat. No. 103138), CD4 (APC/Cy7, clone E-AB-F1353UJ, Elabscience, Cat. No. AF18028), CD8α (PerCP/Cy5.5, clone E-AB-F1104UJ, Elabscience, Cat. No. AF19856), PD-1 (APC, clone 29F.A12 Biolegend, Cat. No. 135210), CD69 (PE/Cy7, clone E-AB-F1187UH, Elabscience, Cat. No. AF19854), TNF-α (BV421, clone MP6-XT22, Biolegend, Cat. No. 506328), FOXP3 (PE, clone FJK-16s, eBioscience, Cat. No. 12-5773-82), p-ZAP70 (PE, clone n3kobu5, eBioscience, Cat. No. 12-9006-41), Perforin (APC, clone S16009A, Biolegend, Cat. No. 154303) and Granzyme B (APC, clone QA16A02, Biolegend, Cat. No. 372203). For human samples, antibodies against CD4 (PerCP/Cy5.5, clone RPA-T4, Biolegend, Cat. No. 300530; APC/Cy7, clone OKT4, Biolegend, Cat. No. 317418), CD19 (APC/Cy7, clone SJ25C1, Biolegend, Cat. No. 363009), IFN-γ (PE, clone 4S.B3, Biolegend, Cat. No. 502509), IL-9 (PE, clone

MH9A4, Biolegend, Cat. No. 507605, PE/Cy7, clone MH9A4, Biolegend, Cat. No. 507612), p-ZAP70 (PE, clone n3kobu5, eBioscience, Cat. No. 12-9006-41), TNFα (PE, clone Mab11, Biolegend, Cat. No. 502908), Perforin (APC, clone dG9, Biolegend, Cat. No. 308111), and Granzyme B (APC, clone QA16A02, Biolegend, Cat. No. 372203) were used.

Flow cytometric acquisition was performed using a BD Fortessa or Cytek Aurora cytometer. Data were analyzed with FlowJo software (v10.8.1 or newer). Fluorescence compensation, isotype-matched controls, and fluorescence-minus-one (FMO) controls were applied as appropriate for multicolor panel validation.

Western Blotting

Cells were washed twice with PBS and lysed in 1 × lysis buffer (NCM, Cat. No. WB3100) supplemented with a protease inhibitor cocktail (Solarbio, Cat. No. P1260). Protein lysates were separated by SDS-polyacrylamide gel electrophoresis (SDS-PAGE; YEASEN, Cat. No. 20325ES62) and transferred to PVDF membranes for immunoblotting.

Membranes were probed with the following primary antibodies: ERK1/2 (clone W15133B, BioLegend, Cat. No. 686902), phospho-ERK1/2 (clone 6B8B69, BioLegend, Cat. No. 369502), MEK1/2 (clone L38C12, Cell Signaling Technology, Cat. No. 4694S), phospho-MEK1/2 (clone 41G9, Cell Signaling Technology, Cat. No. 9154S), SREBP1 (HUABIO, Cat. No. ER1917-19), ZAP70 (Abways, Cat. No. CY6937), NF-κB1 p105/p50 (Proteintech, Cat. No. 14220-1-AP), NF-κB p65 (Proteintech, Cat. No. 10745-1-AP), Total Akt (Cell Signaling Technology, Cat. No. 4691T), phospho-Akt (Poly6490, BioLegend, Cat. No. 649001), mTOR (BioLegend, 6H9B10, Cat. No. 659201), phospho-mTOR (PT0498R, Immunoway, Cat. No. YM8326), α-actinin (Beyotime, Cat. No. AG1022), β-actin (Cell Signaling Technology, Cat. No. 4967), and GAPDH (clone A531, Bioworld, Cat. No. AP0066). HRP-conjugated secondary antibodies included goat anti-rabbit IgG (H+L) (YEASEN, Cat. No. 33101ES60), goat anti-rat IgG(H+L) (proteintech, Cat. No. SA00001-15) and goat anti-mouse IgG (H+L) (ZEN-BIOSCIENCE, Cat. No. 511103).

Molecular weight references were provided by PageRuler Prestained Protein Ladder (Thermo Fisher, Cat. No. 26616) and Ncm Color Marker (NCM, Cat. No. P9006). Protein bands were visualized using enhanced chemiluminescence (ECL) and quantified using ImageJ software.

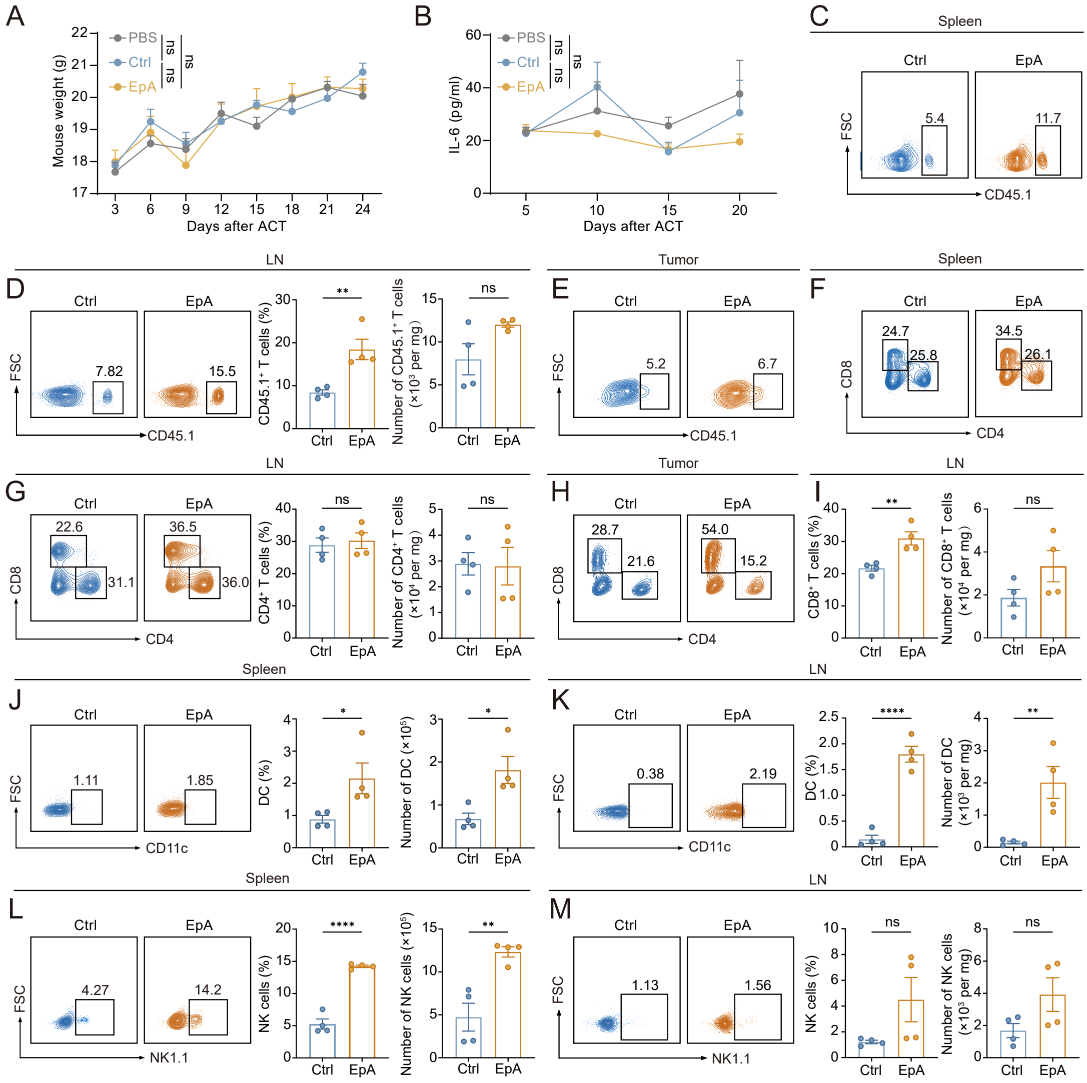

Figure S1. EpA enhances immune responses.

(A) Body weight of mice after ACT in PBS, control, and EpA-treated groups. **(B)** Serum IL-6 levels after ACT, measured by ELISA on days 5, 10, 15, and 20. **(C–E)** Representative flow-cytometric plots showing transferred CD45.1^{+} OT-II Th9 cells in spleens **(C)**, tumor-draining lymph nodes (LNs) **(D)**, and tumors **(E)**; frequencies and absolute numbers of CD45.1^{+} OT-II Th9 cells in LNs are quantified in **(D). (F–I)** Representative flow-cytometric plots of endogenous CD4^{+} and CD8^{+} T cells in spleens **(F)**, LNs **(G)**, and tumors **(H)**; frequencies and absolute numbers of endogenous CD4^{+} **(G)** and CD8^{+} **(I)** T cells in LNs are quantified. **(J and K)** Representative plots, frequencies, and total numbers of dendritic cells (DCs) in spleens **(J)** and LNs **(K). (L and M)** Representative plots, frequencies, and total numbers of natural killer (NK) cells in spleens **(L)** and LNs **(M)**. Data in **(A–M)** are shown as mean ± SEM from $n = 4$ mice per group. Statistical analysis was performed using two-way ANOVA with Tukey's post hoc test **(A),** one-way ANOVA with Tukey's post hoc test **(B)** or two-tailed unpaired Student's t-test **(D** and **G–M)**. ns, not significant. $*p < 0.05$, $**p < 0.01$, $****p < 0.0001$.

A

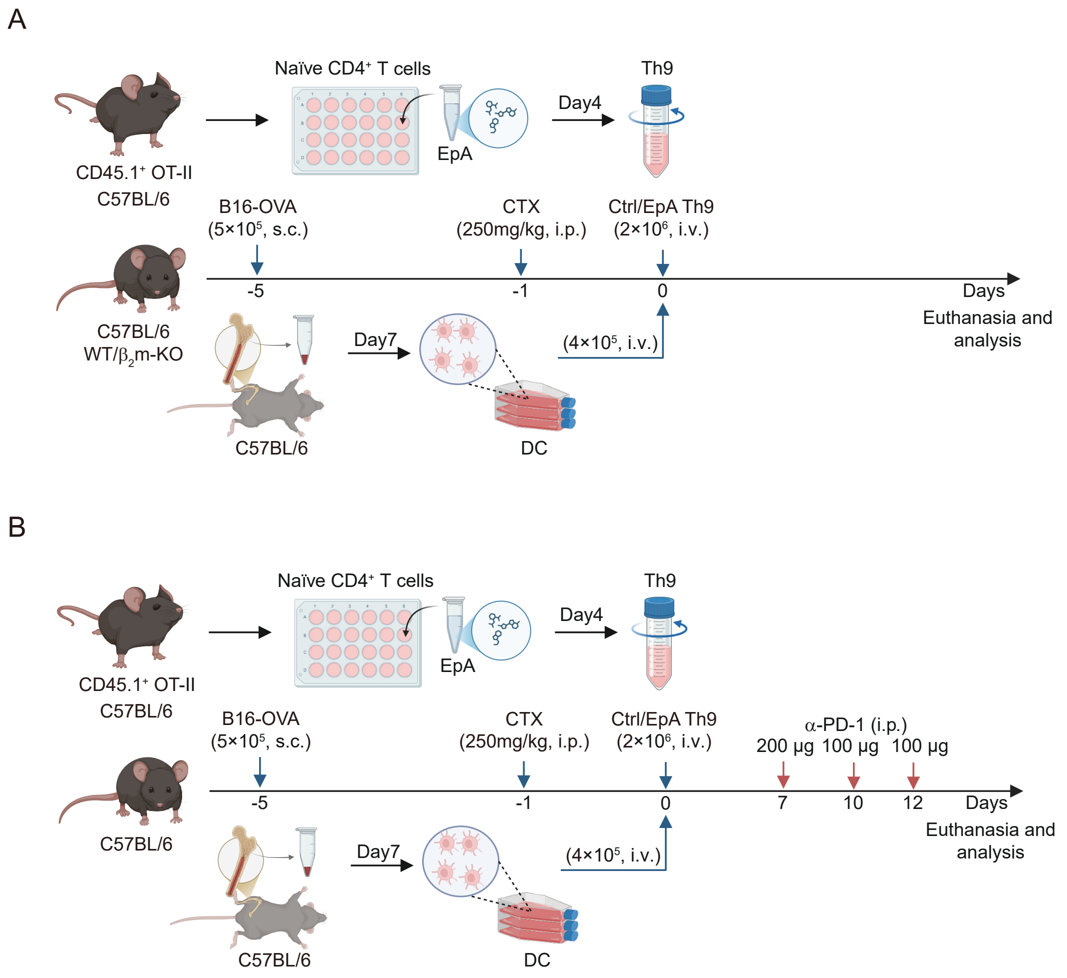

Figure S2. Schematic diagrams of ACT models used in vivo studies.

(A) Diagram of the ACT model in CD8-deficient hosts. C57BL/6 or β2m-KO mice were subcutaneously inoculated with B16-OVA melanoma cells (5×10^5) and preconditioned with CTX on day 4. On day 5, mice were intravenously administered PBS, OT-II Th9 cells (Ctrl), or OT-II EpA-Th9 cells (EpA) (2×10^6) ($n = 4$ mice per group). **(B)** Diagram of the ACT model combined with immune checkpoint blockade. B16-OVA tumor–bearing C57BL/6 mice were treated with ctrl or EpA-induced OT-II Th9 cells (2×10^6, i.v., day 5), followed by intraperitoneal administration of anti–PD-1 antibody (200 μg/mice, i.p.) on day 7, and (100 μg/ mice, i.p.) on day 10 and 12 (n = 6 mice per group). Schematic in (**A**) and (**B**) created using BioRender (https://Biorender.com)

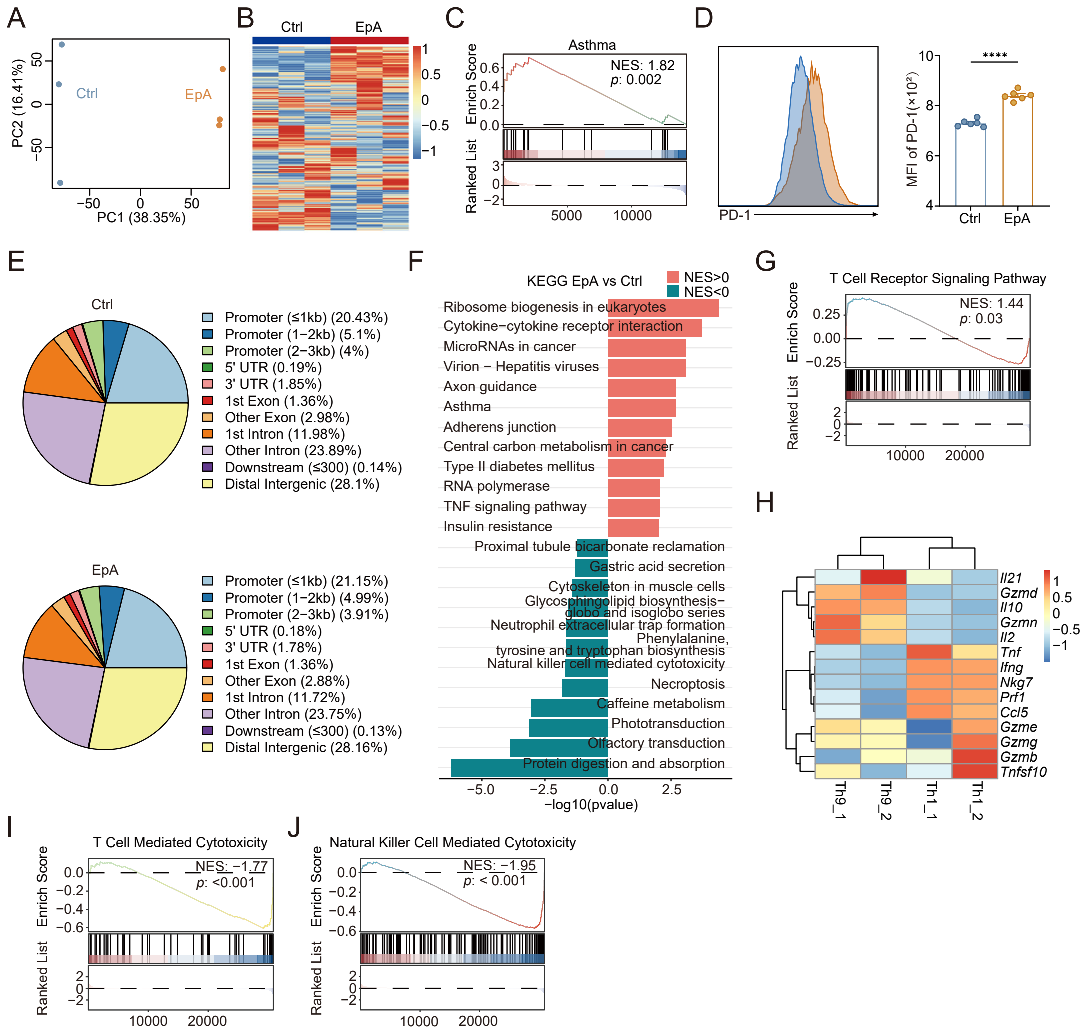

Figure S3. Transcriptomic and phenotypic characterization of EpA-Th9 cells. **(A)** Principal component analysis (PCA) of bulk RNA-seq data showing distinct transcriptional profiles of control and EpA-treated Th9 cells. **(B)** Heatmap of gene expression in RNA-seq data of control and EpA-treated Th9 cells. **(C)** GSEA of asthma pathway. **(D)** Flow cytometric analysis of PD-1 expression (mean ± SEM, $n = 6$). ****$p < 0.0001$. **(E)** Distribution of ATAC-seq peaks across genomic regions. **(F)** KEGG pathway enrichment analysis of different pathways between control and EpA-Th9 groups. Red bars indicate pathways upregulated in EpA-Th9 cells; green bars indicate downregulated pathways. **(G–J)** Data reanalysis of GEO dataset (accession number: GSE97087). GSEA of T cell receptor signaling pathway in Th9 compared to Th1 cells **(G)**. Heatmap of cytotoxic effector genes and cytokines **(H)**. GSEA plots showing T cell–mediated cytotoxicity pathway **(I)** and natural killer cell–mediated cytotoxicity pathway **(J)**. Statistical analysis was performed using two-tailed unpaired Student’s t-test **(D)**.

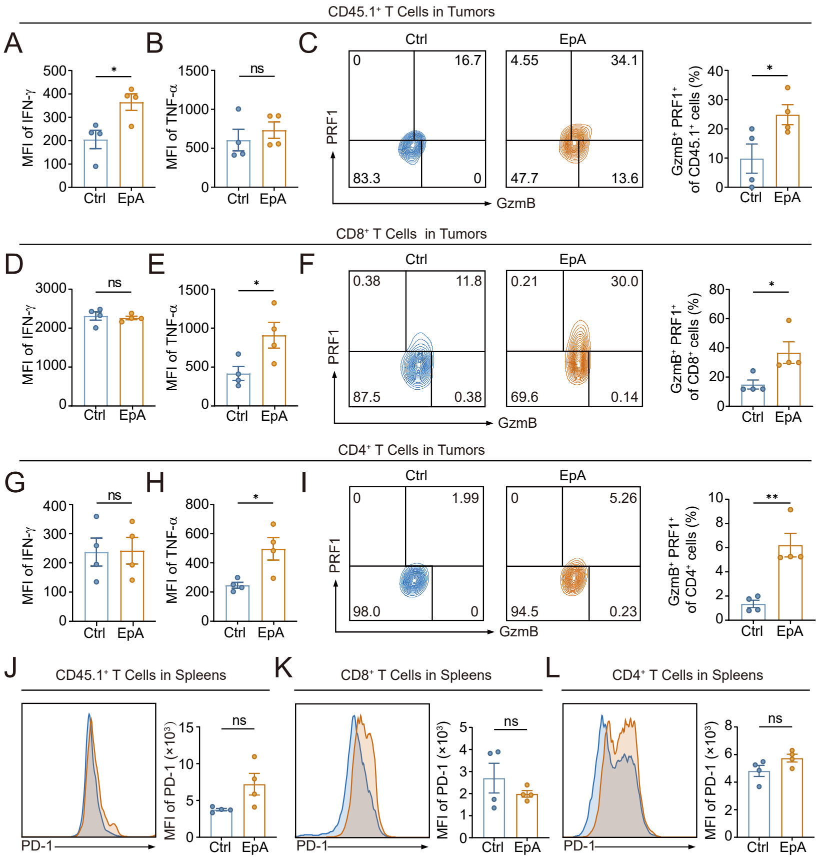

Figure S4. EpA enhances cytotoxic effector functions of tumor-infiltrating Th9 cells and endogenous T cells.

(A–C) Mean fluorescence intensity (MFI) of IFN-γ **(A)** and TNF-α **(B)**, and representative flow-cytometric plots with quantification of Granzyme B⁺ Perforin⁺ cells **(C)** among adoptively transferred CD45.1⁺ Th9 cells in tumors. **(D–F)** MFI of IFN-γ **(D)** and TNF-α **(E)**, and representative plots with quantification of Granzyme B⁺ Perforin⁺ cells **(F)** among tumor-infiltrating CD8⁺ T cells. **(G–I)** MFI of IFN-γ **(G)** and TNF-α **(H)**, and representative plots with quantification of Granzyme B⁺ Perforin⁺ cells **(I)** among tumor-infiltrating CD4⁺ T cells. **(J–L)** PD-1 expression in adoptively transferred CD45.1⁺ Th9 cells **(J)**, endogenous CD8⁺ T cells **(K)**, and endogenous CD4⁺ T cells **(L)** from spleens of tumor-bearing mice. Data in **(A–L)** are shown as mean ± SEM from $n = 4$ mice per group. Statistical analysis was performed using two-tailed unpaired Student's t-test **(A–L)**. ns, not significant. *$p < 0.05$, **$p < 0.01$.

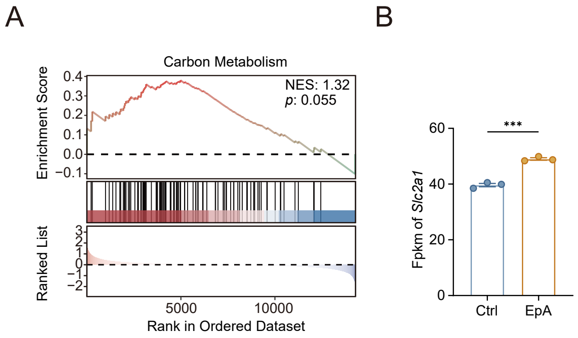

Figure S5. EpA promotes metabolic reprogramming in Th9 cells.
(A) GSEA of carbon metabolism pathway. **(B)** Fpkm of *Slc2a1* (GLUT1) in control and EpA-treated Th9 cells (mean ± SEM, $n = 3$). Statistical analysis was performed using two-tailed unpaired Student's t-test. ***$p < 0.001$.

A

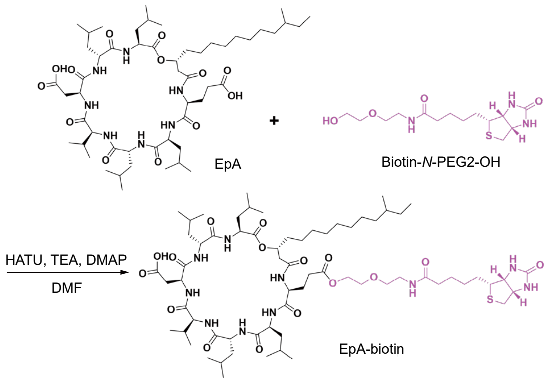

B

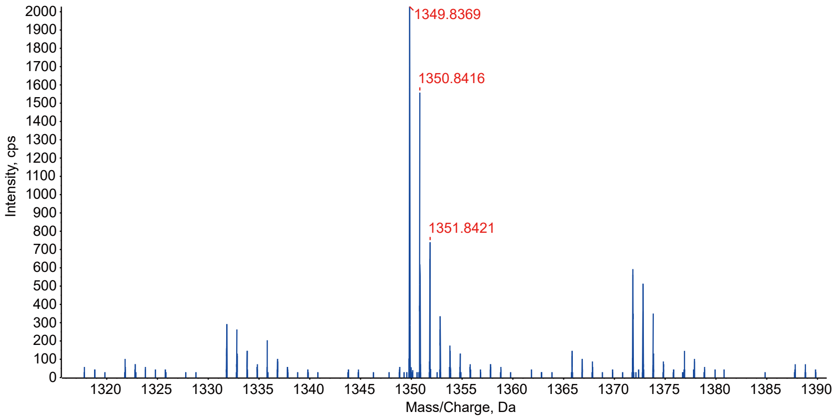

Hit	Formula	*m/z*	RDB	ppm	MS Rank
1	$C_{67}H_{116}N_{10}O_{16}S$	1349.8364	15.0	0.5	1

Figure S6. Chemical synthesis of molecular probe (biotin-conjugated EpA) and its mass spectrometry validation.

(A) Synthetic scheme for biotinylated EpA. **(B)** LC–MS spectrum of the biotin-conjugated EpA product, showing a dominant peak at *m/z* = 1349.8369 $[M+H]^+$, confirming successful conjugation of the biotin moiety.

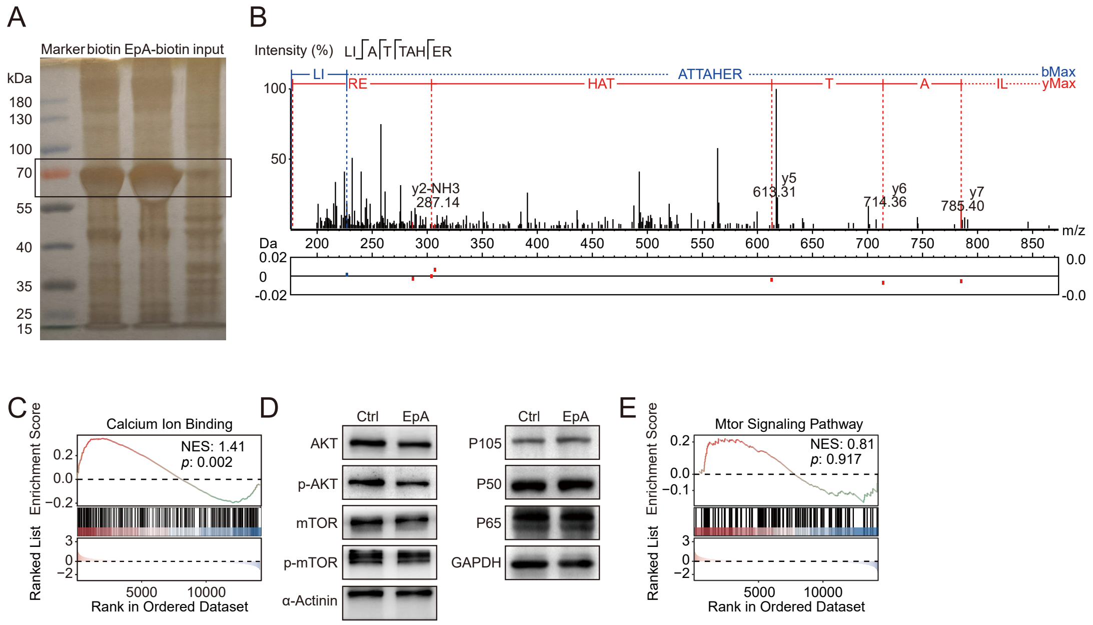

Figure S7. Identification of EpA-binding proteins and analysis of downstream signaling pathways.

(A) Affinity-based pull-down assay using biotin-conjugated EpA and streptavidin magnetic beads in Th9 cell lysates. EpA-associated proteins were visualized by silver staining. **(B)** Representative mass spectrometry spectrum showing the identification of a ZAP70-derived peptide among EpA-bound proteins. **(C)** GSEA showing enrichment of calcium binding in EpA-treated cells. **(D)** Immunoblot analysis of total and phosphorylated AKT, mTOR, and NF-κB signaling components (P105, P50, and P65) in control and EpA-treated Th9 cells. **(E)** GSEA showing mTOR signaling pathway.

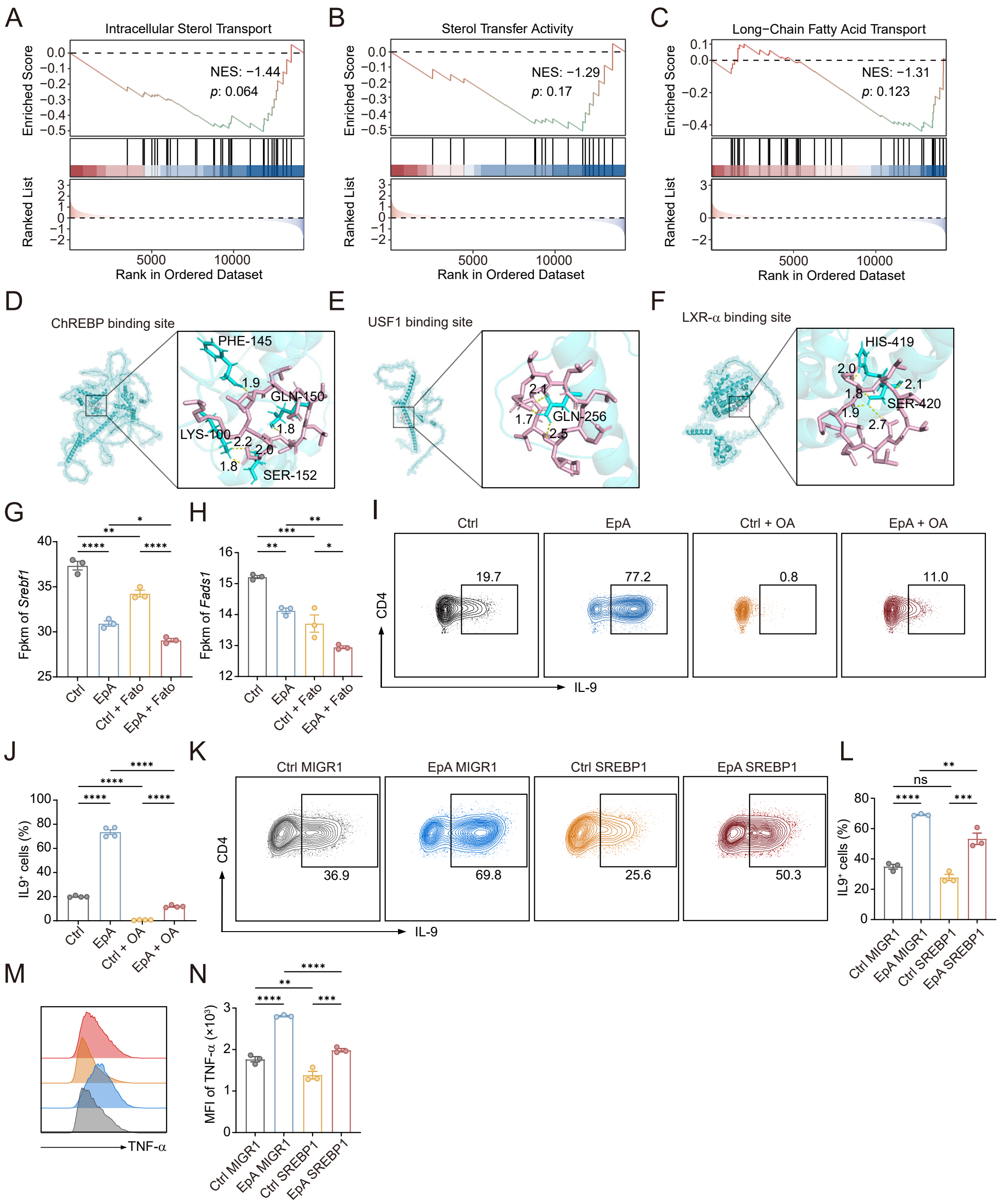

Figure S8. EpA-associated modulation of lipid metabolism and its impact on Th9 cell function.

(A–C) GSEA of RNA-seq data showing intracellular sterol transport **(A)**, sterol transfer activity **(B)**, and long-chain fatty acid transport **(C)** pathways in control and EpA-treated Th9 cells. **(D–F)** Predicted molecular docking models of EpA (pink) with key lipid metabolism–related transcription factors, including ChREBP **(D)**, USF1 **(E)**, and LXR-α **(F)** (proteins shown in blue). **(G and H)** RNA-seq quantification of *Srebf1* **(G)** and *Fads1* **(H)** transcript levels in Th9 cells treated with vehicle, EpA (10 μM), Fatostatin (5 μM), or the combination ($n = 3$). **(I and J)** Flow-cytometric analysis of intracellular IL-9 expression in Th9 cells treated with control, EpA (10 μM), oleic acid (OA, 50 μM), or both, showing representative plots **(I)** and quantification of IL-9^{+} cells **(J)** ($n = 4$). **(K and L)** Flow-cytometric analysis of IL-9 expression in Th9 cells transduced with either control (MIGR1) or SREBP1-

overexpressing vectors, with or without EpA treatment, showing representative plots **(K)** and quantification of IL-9$^+$ cells **(L)** ($n = 3$). **(M** and **N)** Flow-cytometric analysis of intracellular TNF-α expression in Th9 cells treated as in **(K)**, showing representative histograms **(M)** and quantification of mean fluorescence intensity **(N)** ($n = 3$). Statistical analysis was performed using one-way ANOVA followed by Tukey's post hoc test (**G** and **H**, **J**, **L**, **N**). ns, not significant. Repeated data are presented as mean ± SEM. *$p < 0.05$, **$p < 0.01$, ***$p < 0.001$, ****$p < 0.0001$.

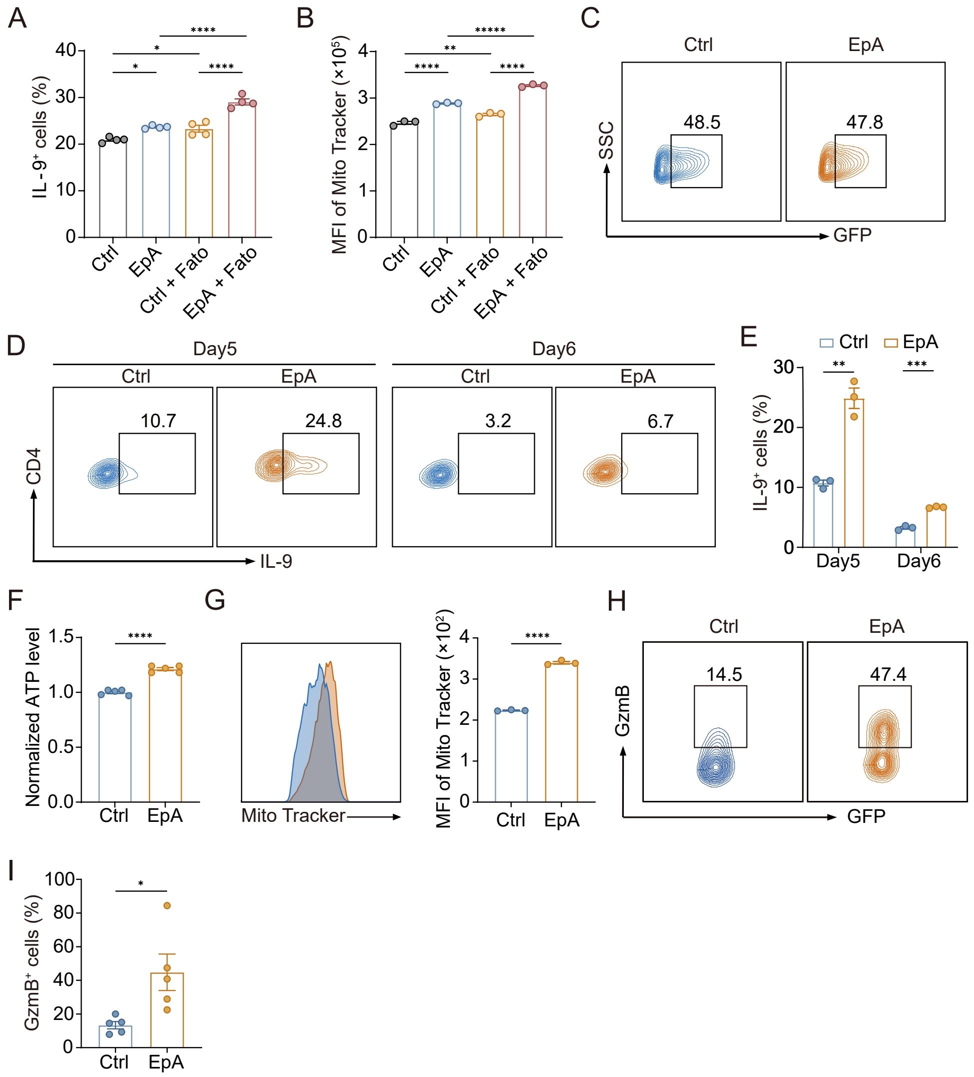

Figure S9. Effects of EpA on human Th9 CAR-T cells.

(A) Flow-cytometric analysis of intracellular IL-9 expression in human Th9 cells treated with EpA, Fatostatin, or both (n = 4). **(B)** Flow-cytometric assessment of mitochondrial mass using Mito Tracker in human Th9 cells treated as in **(A)** (n = 3). **(C)** Flow-cytometric analysis of infection efficiency in Th9 cells transduced with a CD19-directed CAR lentivirus. **(D and E)** Flow-cytometric analysis of IL-9 expression in human Th9 CAR-T cells on days 5 and 6 of in vitro differentiation, showing representative plots **(D)** and quantification **(E)** (n = 3). **(F)** Measurement of intracellular ATP levels normalized to cell number in human Th9 CAR-T cells (n =5). **(G)** Flow-cytometric analysis of mitochondrial mass using Mito Tracker in human Th9 CAR-T cells (n = 3). **(H and I)** Flow-cytometric analysis of granzyme B$^+$ CAR-Th9 cells isolated from spleens after adoptive transfer, showing representative plots **(H)** and quantification **(I)** (n = 5 mice per group). Statistical analysis was performed using one-way ANOVA with Tukey's post hoc test (A and B), or two-tailed unpaired Student's t-test (E–G, and I). Repeated data are presented as mean ± SEM. *p < 0.05, **p < 0.01, ***p < 0.001, ****p < 0.0001.

Table 1. Information of metabolites and their effect on Th9 differentiation.

Part I. The chemical structures of metabolites **1-65**.

Cyclopeptides

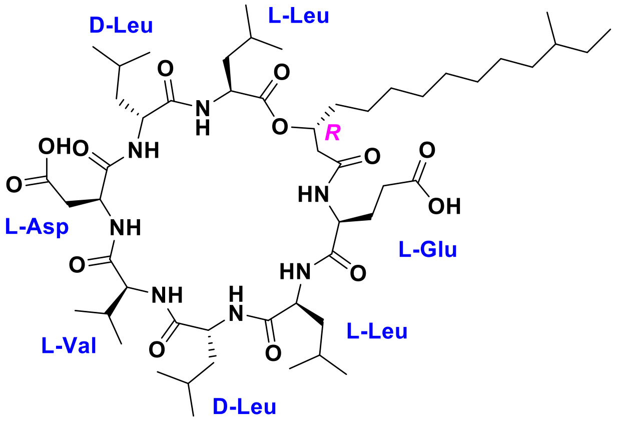

EpA (1)

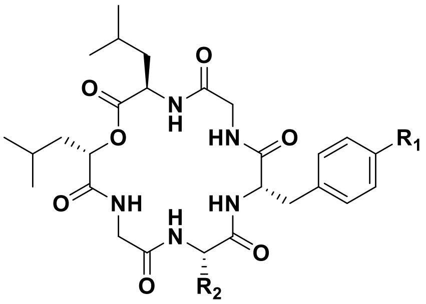

2 R_1 = H R_2 = $CH(CH_3)_2$
3 R_1 = OH R_2 = $CH(CH_3)_2$

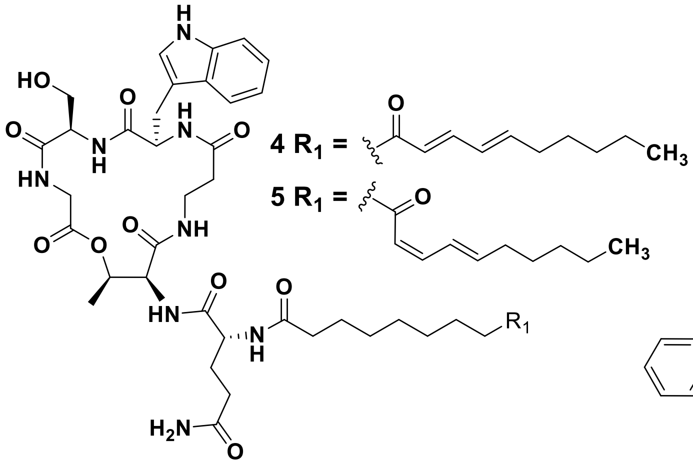

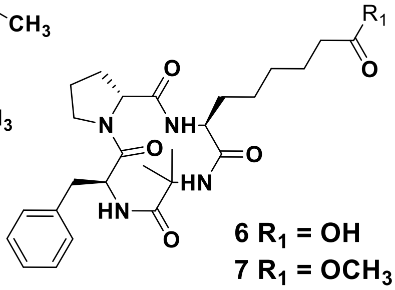

6 R_1 = OH
7 R_1 = OCH_3

Terpenoids

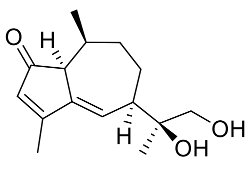

8

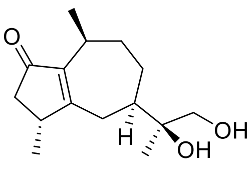

9

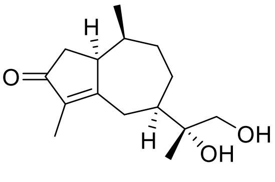

10

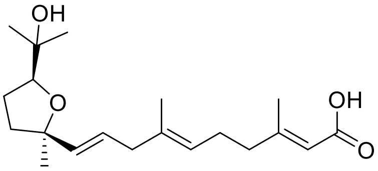

11

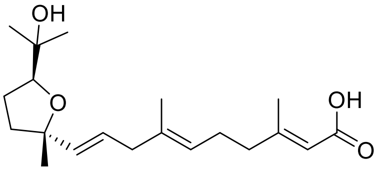

12

Cyclodipeptides

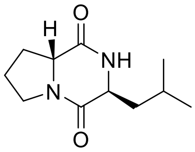

13

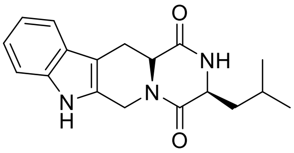

14

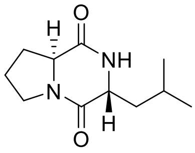

15

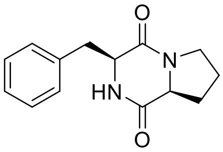

16

Alkaloids

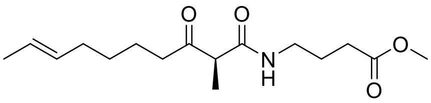

17

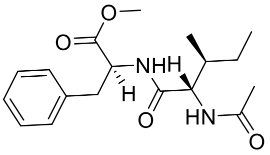

18

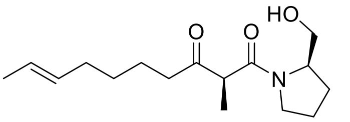

19

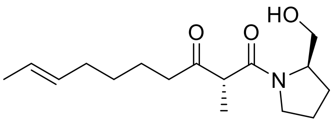

20

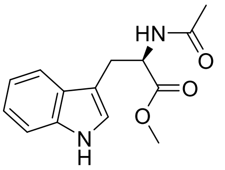

21

Sterols

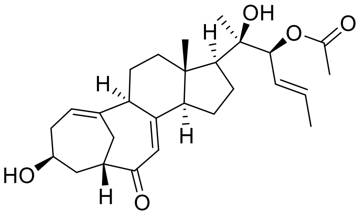

22

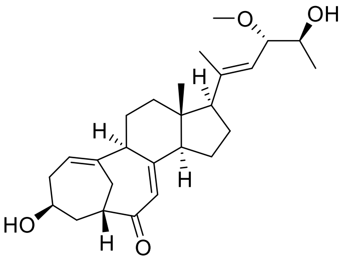

23

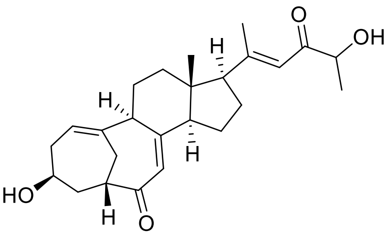

24

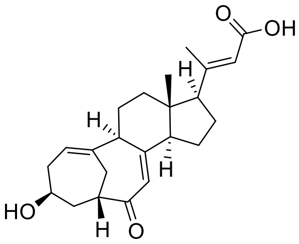

25

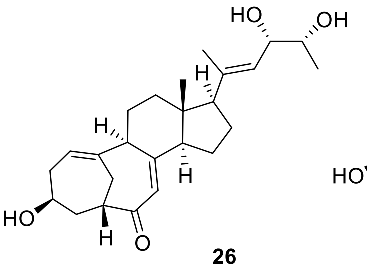

26

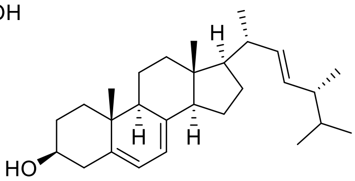

27

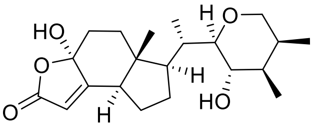

28

Decalins

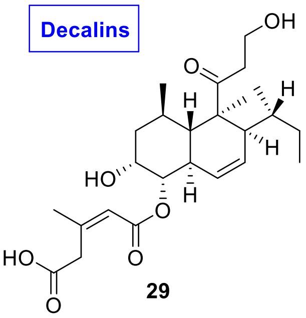

29

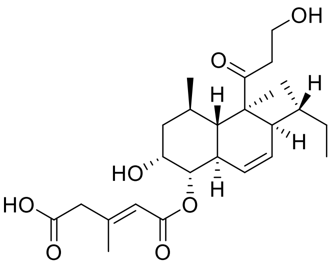

30

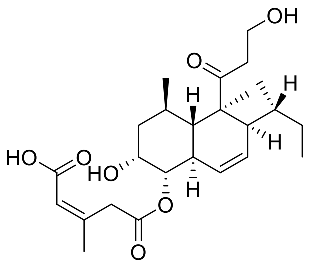

31

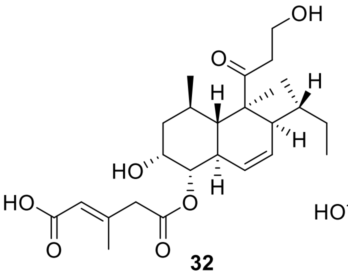

32

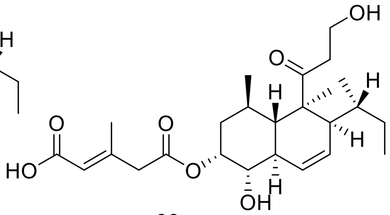

33

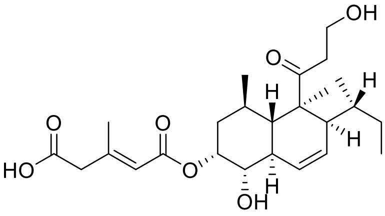

34

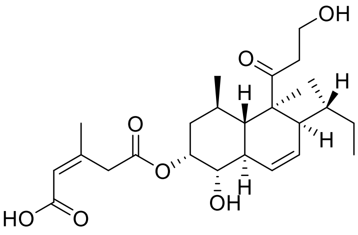

35

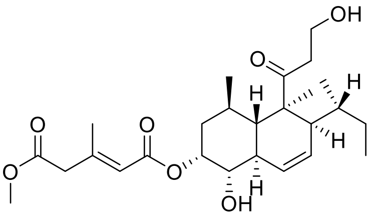

36

Citrinins

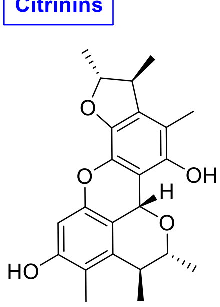

37

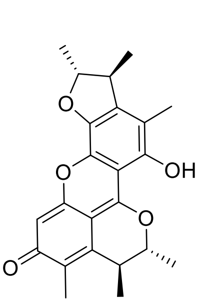

38

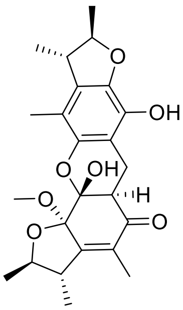

39

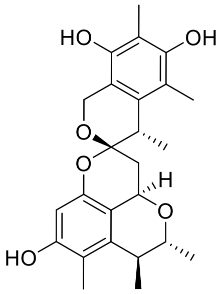

40

Aromatics

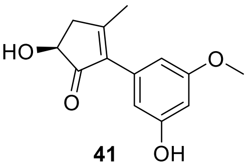

41

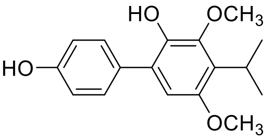

42

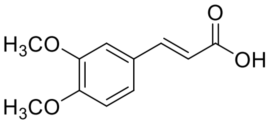

43

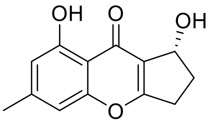

44

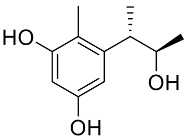

45

Isocoumarins

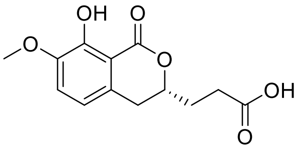

46

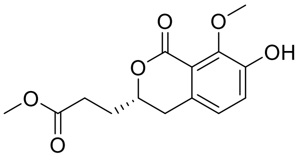

47

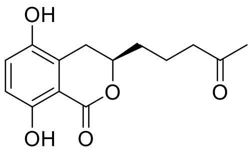

48

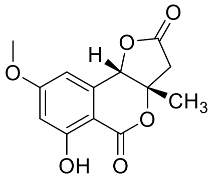

49

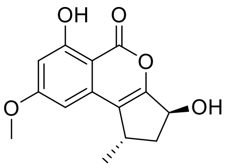

50

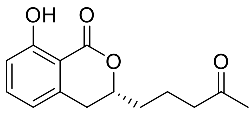

51

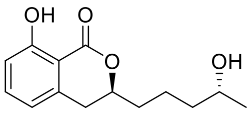

52

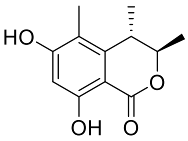

53

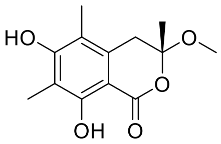

54

Phthalides

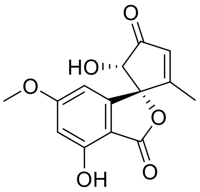

55

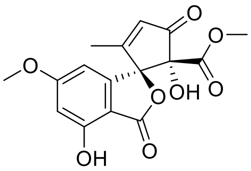

56

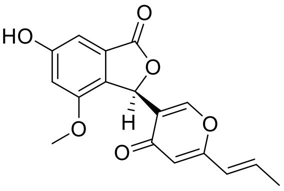

57

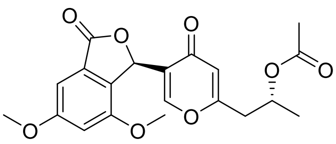

58

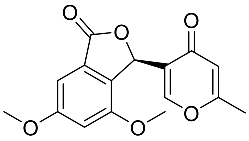

59

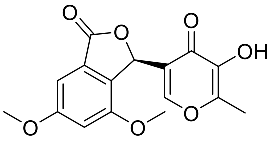

60

Azaphilones

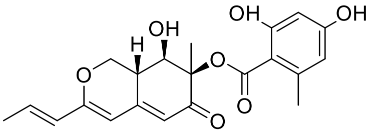

61

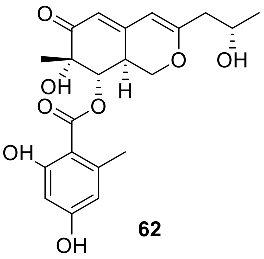

62

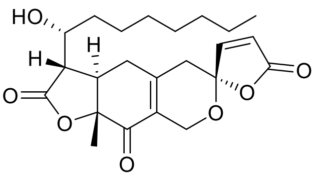

63

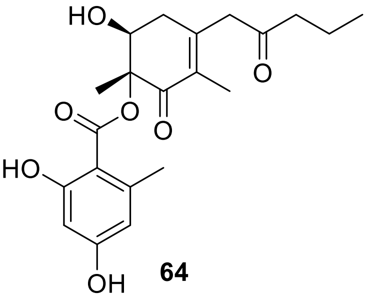

64

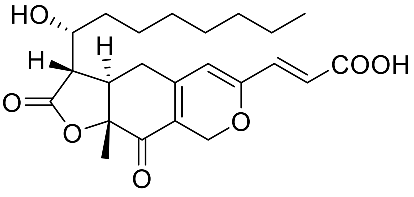

65

Part II. Detailed information of metabolites and their effects on Th9 differentiation.

Name	Fold change of IL-9 expression	Origins	Hosts
EpA (**1**)	4.34	*Endomelanconiopsis sp.*	*Ganoderma lucidum*
1962B (**2**)	1.92	*Trichoderma sp.*	*Ganoderma lucidum*
1962A (**3**)	2.22	*Trichoderma sp.*	*Ganoderma lucidum*
Aselacin C (**4**)	2.13	*Trichoderma sp.*	*Ganoderma lucidum*
Aselacin derivative (**5**)	2.98	*Trichoderma sp.*	*Ganoderma lucidum*
Chlamydocin derivative (**6**)	0.91	*Trichoderma sp.*	*Ganoderma lucidum*
Tirchocyclopeptide B (**7**)	0.026	*Trichoderma sp.*	*Ganoderma lucidum*
Trichodermanol B (**8**)	2.95	*Trichoderma sp.*	*Ganoderma lucidum*
Trichodermanol C (**9**)	3.06	*Trichoderma sp.*	*Ganoderma lucidum*
(1*S*,7*R*,10*S*,11*R*)-3-oxoguai-4-ene-11,12-diol (**10**)	1.98	*Trichoderma sp.*	*Ganoderma lucidum*
Mucorinic acid A (**11**)	2.42	*Mucor irregularis*	*Ganoderma lucidum*
Mucorinic acid B (**12**)	3.70	*Mucor irregularis*	*Ganoderma lucidum*
Cyclo-((*S*)-Pro-(*R)*-Leu) (**13**)	2.54	*Talaromyces sp.*	*Ganoderma lucidum*
(3*S*,12a*S*)-2,3,6,7,12,12a-Hexahydro-3-(2-methylpropyl)pyrazino[1′	1.65	*Talaromyces sp.*	*Ganoderma lucidum*

,2′:1,6]pyrido[3,4-*b*]indole-1,4-dione (**14**)			
Cyclo-((*S*)-Pro-(*R*)-Leu) (**15**)	2.50	*Trichoderma sp.*	*Ganoderma lucidum*
(3*S*,8a*S*)-3-Benzylhexahydropyrrolo [1,2-a] pyrazine-1,4-dione (**16**)	2.66	*Mucor irregularis*	*Ganoderma lucidum*
Ethyl 2-(2-acetamido-3-methylpentanamido)-3-phenylpropanoate (**17**)	2.35	*Nigrospora oryzae*	*Periplaneta americana*
Methyl (2*S*,8*E*)-1′-(2-methyl-3-oxodec-8-enamido) butanoate (**18**)	2.15	*Nigrospora oryzae*	*Periplaneta americana*
(*R*)-scalusamide A (**19**)	2.53	*Nigrospora oryzae*	*Periplaneta americana*
(*S*)-scalusamide A (**20**)	2.74	*Nigrospora oryzae*	*Periplaneta americana*
N-acetyltryptophan methyl ester (**21**)	3.15	*Nigrospora oryzae*	*Periplaneta americana*
22-acetylisocyclocitrinol A (**22**)	1.57	*Nigrospora oryzae*	*Periplaneta americana*
Erythro-23-O-methylneocyclocitrinol (**23**)	1.10	*Nigrospora oryzae*	*Periplaneta americana*
23-oxoneocyclocitrinol (**24**)	1.95	*Nigrospora oryzae*	*Periplaneta americana*
Norcyclocitrinoicacid A (**25**)	3.40	*Nigrospora oryzae*	*Periplaneta americana*
Neocyclocitrinol D (**26**)	0.76	*Mucor irregularis*	*Ganoderma lucidum*
Ergosta-5,7,22-trien-3*β*-ol (**27**)	3.19	*Nigrospora oryzae*	*Periplaneta americana*
Trichodermanol A (**28**)	1.25	*Trichoderma sp.*	*Ganoderma lucidum*
Tandyukisin J (**29**)	1.18	*Trichoderma harzianum*	*Ganoderma lucidum*
Tandyukisin I (**30**)	1.318	*Trichoderma harzianum*	*Ganoderma lucidum*
Tandyukisin C (**31**)	1.228	*Trichoderma harzianum*	*Ganoderma lucidum*
Tandyukisin H (**32**)	1.148	*Trichoderma harzianum*	*Ganoderma lucidum*
Tandyukisin G (**33**)	1.06	*Trichoderma harzianum*	*Ganoderma*

			lucidum
Trichoharzin (**34**)	1.38	*Trichoderma harzianum*	*Ganoderma lucidum*
Tandyukisin D (**35**)	1.17	*Trichoderma harzianum*	*Ganoderma lucidum*
Trichoharzin B (**36**)	1.16	*Trichoderma harzianum*	*Ganoderma lucidum*
Penicitrinol A (**37**)	1.09	*Mucor irregularis*	*Ganoderma lucidum*
Penicitrinone A （**38**）	1.08	*Nigrospora oryzae*	*Periplaneta americana*
Penidicitrinin A （**39**）	1.11	*Nigrospora oryzae*	*Periplaneta americana*
Xerucitrinin A (**40**)	1.07	*Nigrospora oryzae*	*Periplaneta americana*
Alternariphent A (**41**)	0.89	*Talaromyces sp.*	*Ganoderma lucidum*
3,5-Dimethoxy-4-(1-methylethyl)[1,1′-biphenyl]-2,4′-diol (**42**)	1.06	*Talaromyces sp.*	*Ganoderma lucidum*
Trans-3,4-dimethoxycinnamic acid (**43**)	1.19	*Talaromyces sp.*	*Ganoderma lucidum*
Coniochaetone B (**44**)	1.22	*Nigrospora oryzae*	*Periplaneta americana*
Phenol A (**45**)	0.92	*Nigrospora oryzae*	*Periplaneta americana*
Isocoumarin (**46**)	1.24	*Talaromyces sp.*	*Ganoderma lucidum*
Peniciisocoumarin E (**47**)	1.43	*Talaromyces sp.*	*Ganoderma lucidum*
Penicilloxalone B (**48**)	1.50	*Talaromyces sp.*	*Ganoderma lucidum*
(3a*S*,9b*S*)-3a,9b-Dihydro-6-hydroxy-8-methoxy-3a-methyl-2*H*-furo[3,2-*c*][2]benzopyran-2,5(3*H*)-dione (**49**)	1.34	*Talaromyces sp.*	*Ganoderma lucidum*
Phialophoriol (**50**)	0.04	*Talaromyces sp.*	*Ganoderma lucidum*
Aspergillumarins A (**51**)	1.52	*Talaromyces sp.*	*Ganoderma lucidum*
Isocoumarin derivatives (**52**)	1.24	*Talaromyces sp.*	*Ganoderma lucidum*

(3*R*,4*S*)-6,8-dihydroxy-3,4,5-trimethy Lisocoumarin (**53**)	1.25	*Nigrospora oryzae*	*Periplaneta americana*
Stoloniferol A (**54**)	1.27	*Nigrospora oryzae*	*Periplaneta americana*
Talaroflavone (**55**)	1.56	*Talaromyces sp.*	*Ganoderma lucidum*
Alternaone A (**56**)	1.18	*Talaromyces sp.*	*Ganoderma lucidum*
6-Demethylvermistatin (**57**)	1.28	*Talaromyces sp.*	*Ganoderma lucidum*
Dihydrovermistatin (**58**)	1.29	*Talaromyces sp.*	*Ganoderma lucidum*
Penisimplicissin (**59**)	1.09	*Talaromyces sp.*	*Ganoderma lucidum*
(3*R*)-3-(5-hydroxy-6-methyl-4-oxo-4*H*-pyran-3-yl)-4,6-dimethoxy-1(3*H*)-isobenzofuranone (**60**)	1.28	*Talaromyces sp.*	*Ganoderma lucidum*
Pinophilins A (**61**)	1.18	*Talaromyces sp.*	*Ganoderma lucidum*
Pinophilins E (**62**)	1.30	*Talaromyces sp.*	*Ganoderma lucidum*
Sequoiamonascin A (**63**)	0.67	*Talaromyces sp.*	*Ganoderma lucidum*
Talarophilone (**64**)	0.79	*Talaromyces sp.*	*Ganoderma lucidum*
Sequoiamonascin C (**65**)	2.02	*Talaromyces sp.*	*Ganoderma lucidum*

Table 2. Structural characterization of EpA by NMR and high-resolution mass spectrometry.

Part I. ^{1}H (600 MHz) NMR spectrum of EpA in DMSO-d6.

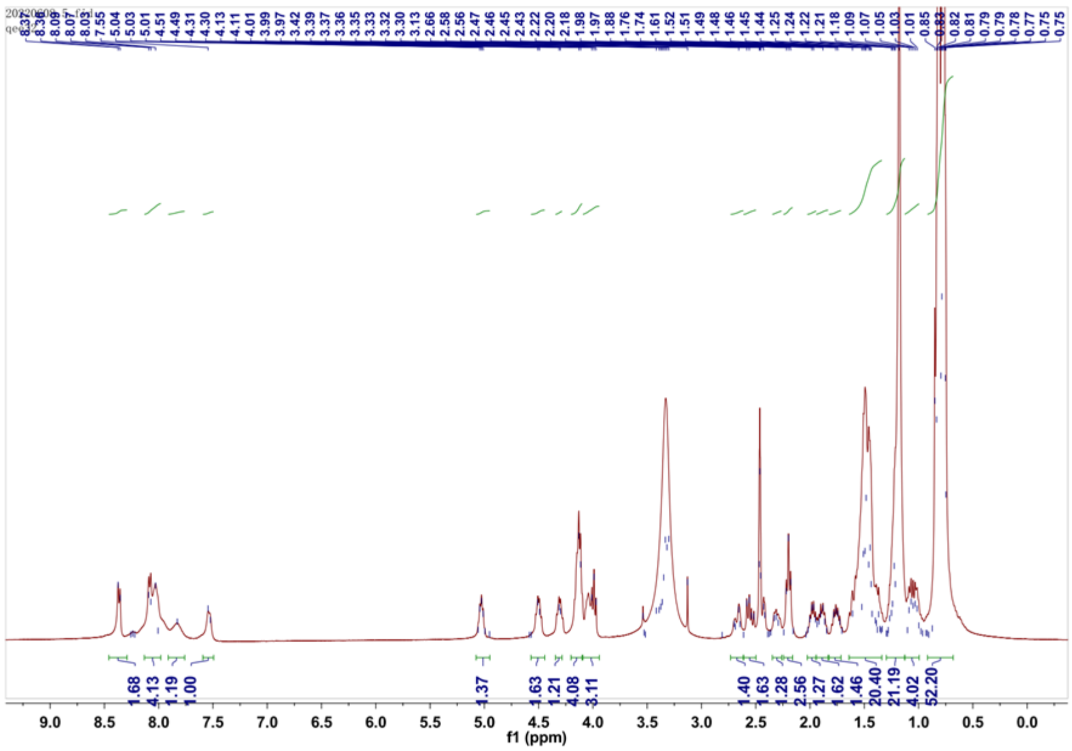

Part II. ^{13}C (150 MHz) NMR spectra of EpA in DMSO-d_6.

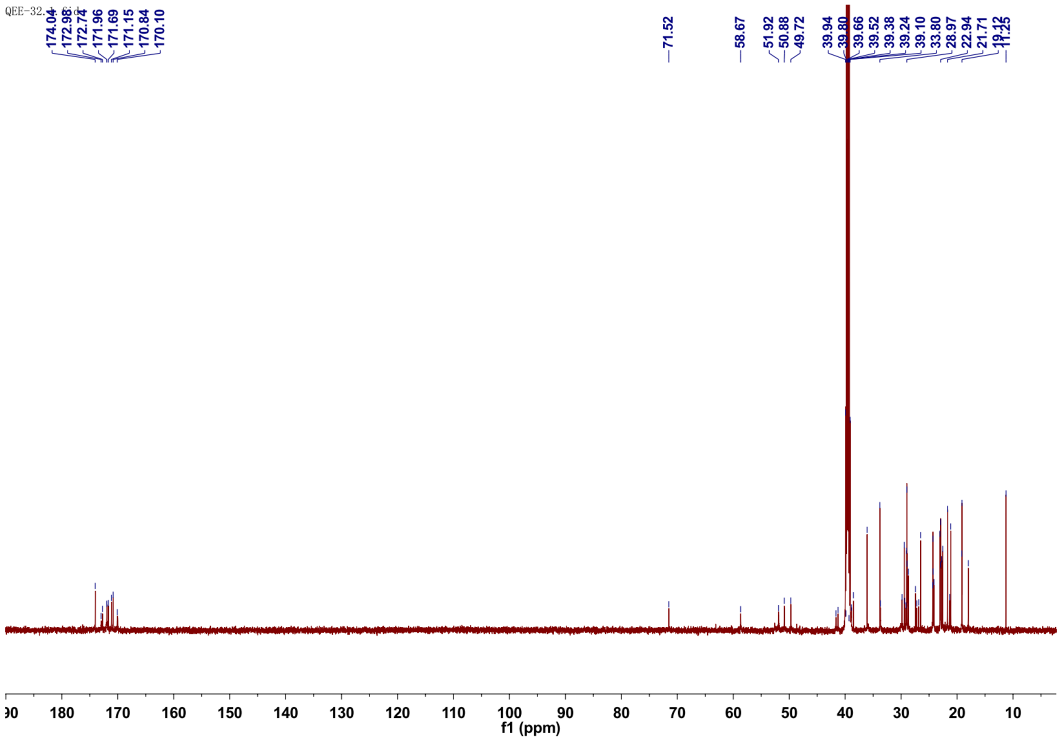

Part III. HRESIMS of compound EpA

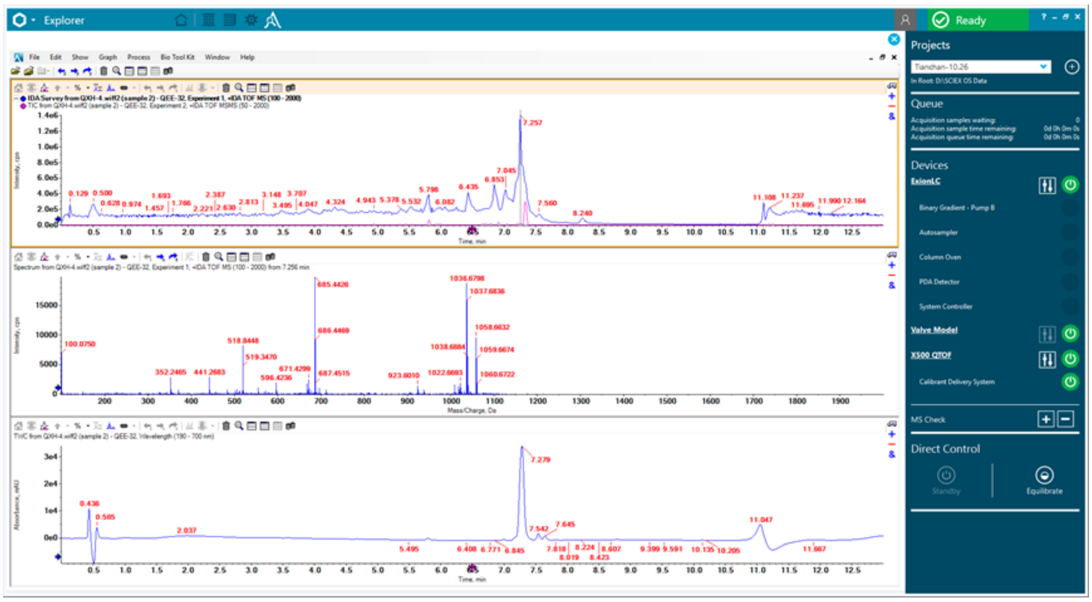

Table 3. Molecular docking predictions of EpA-bound proteins identified by pull-down assay.

EpA-bound proteins identified by pull-down assay	
symbol	score（kcal/mol）
Q14AX6|CDK12_MOUSE	-13.29
Q9QX66|DPF1_MOUSE	-13.19
Q69ZA1|CDK13_MOUSE	-12.3
P43404|ZAP70_MOUSE	-12.17
P17932|RL32P_MOUSE	-11.95
Q3TKT4|SMCA4_MOUSE	-11.86
O35495|CDK14_MOUSE	-11.77
Q05920|PYC_MOUSE	-11.55
Q8K4B0|MTA1_MOUSE	-11.49
Q61210|ARHG1_MOUSE	-11.49
Q924K8|MTA3_MOUSE	-11.27
P63094|GNAS2_MOUSE	-11.16
Q9DB96|NGDN_MOUSE	-11.12
P32883|RASK_MOUSE	-11.07
Q8CGZ0|CHERP_MOUSE	-10.98
Q91ZE5|AGRE4_MOUSE	-10.92
Q6PDG5|SMRC2_MOUSE	-10.83
Q7TPB0|PLPR3_MOUSE	-10.8
Q8K0D0|CDK17_MOUSE	-10.72
Q5S006|LRRK2_MOUSE	-10.58
Q6A068|CDC5L_MOUSE	-10.57
P56716|RP1_MOUSE	-10.51
Q6NZJ6|IF4G1_MOUSE	-10.46
P54823|DDX6_MOUSE	-10.42
Q6DIC0|SMCA2_MOUSE	-10.3
Q99J95|CDK9_MOUSE	-10.29
B1AZI6|THOC2_MOUSE	-10.12
Q9JL26|FMNL1_MOUSE	-10.04
Q8BHD7|PTBP3_MOUSE	-9.9
Q9R0E2|PLOD1_MOUSE	-9.78
Q9D992|MAJIN_MOUSE	-9.78
Q9CWH5|TRM11_MOUSE	-9.78
Q91ZA3|PCCA_MOUSE	-9.77
Q64237|DOPO_MOUSE	-9.74
Q3V3A1|CDK15_MOUSE	-9.71
Q04899|CDK18_MOUSE	-9.67
Q9QXX4|S2513_MOUSE	-9.58

Q04735|CDK16_MOUSE	-9.5
Q99MN9|PCCB_MOUSE	-9.17
P97376|FRG1_MOUSE	-9.15
P97496|SMRC1_MOUSE	-9.12
P04187|GRAB_MOUSE	-9
Q6R0H7|GNAS1_MOUSE	-8.89
Q9Z0N1|IF2G_MOUSE	-8.88
Q9Z0N2|IF2H_MOUSE	-8.59
P61028|RAB8B_MOUSE	-8.55
P70429|EVL_MOUSE	-8.49
Q9ESW4|AGK_MOUSE	-8.48
Q9CZ83|RM55_MOUSE	-8.44
Q61103|REQU_MOUSE	-8.3
Q61411|RASH_MOUSE	-8.12
B2RSH2|GNAI1_MOUSE	-8.07
Q8CGK7|GNAL_MOUSE	-8.01
Q9D937|CK098_MOUSE	-7.98
P58854|GCP3_MOUSE	-7.97
P25799|NFKB1_MOUSE	-7.95
Q9DA69|IFT43_MOUSE	-7.84
P50149|GNAT2_MOUSE	-7.79
P20612|GNAT1_MOUSE	-7.16
Q922B2|SYDC_MOUSE	-7.12
Q3V3I2|GNAT3_MOUSE	-7.01
P08556|RASN_MOUSE	-6.96
Q64261|CDK6_MOUSE	-6.86
Q6ZQ06|CE162_MOUSE	-6.85
Q9DC51|GNAI3_MOUSE	-6.83
Q91YH5|ATLA3_MOUSE	-6.62
P18872|GNAO_MOUSE	-6.35
Q8C9S4|CC186_MOUSE	-5.83
Q99JY9|ARP3_MOUSE	-4.94
O35639|ANXA3_MOUSE	-4.68
P09542|MYL3_MOUSE	-4.51
P05977|MYL1_MOUSE	-2.89
Q9JHJ0|TMOD3_MOUSE	-2.56
Q8CI43|MYL6B_MOUSE	-2.21
P21107|TPM3_MOUSE	-1.32
Q6IRU2|TPM4_MOUSE	-1.2
P58774|TPM2_MOUSE	0
P58771|TPM1_MOUSE	0.28
Q9D7G0|PRPS1_MOUSE	72.56
P49615|CDK5_MOUSE	118.6

P30285|CDK4_MOUSE	173.54
P97377|CDK2_MOUSE	453.35
Q80YP0|CDK3_MOUSE	1896.88
Q3TLS3|GDPP1_MOUSE	5114.64
Q6PHN9|RAB35_MOUSE	7637.55
E9PVX6|KI67_MOUSE	16220.88
Q7TPH6|MYCB2_MOUSE	17124.83

Table 4. Docking energies and expression profiles of lipid-metabolism–related transcription factors.

Receptor Protein	Free energy of binding(kcal/mol)	mRNA expression in Th9 (fpkm)
ChREBP	-11.24	0.02
SREBP1	-9.89	37.34
USF1	-6.89	13.00
LXR-α	-6.83	0.18

Table 5. Quantitative PCR Primers.

Human *SPI1* F	CCCTCAGCCATCAGAAGACC
Human *SPI1* R	CTGGAGCTCCGTGAAGTTGT
Human *IL9* F	GACCAGTTGTCTCTGTTTGGGC
Human *IL9* R	TTTCACCCGACTGAAAATCAGTGG
Human *IRF4* F	AGCCCAGCAGGTTCACAACT
Human *IRF4* R	GCTTCGGCAGACCTTATGCT
Human *GAPDH* F	GGAGTCCACTGGCGTCTTCA
Human *GAPDH* R	TGCAGGAGGCATTGCTGAT
Mouse *Irf4* F	AACTACATGATGCCACCCCA
Mouse *Irf4* R	TATGCTTGGCTCAATGGGGA
Mouse *Gapdh* F	TTGATGGCAACAATCTCCAC
Mouse *Gapdh* R	CGTCCCGTAGACAAAATGGT
Mouse *Batf* F	AGAAGGCTGACAAGCTCCAC
Mouse *Batf* R	ACCGAAGCTGCACAAAGTTC
Mouse *Batf3* F	CAGCAGTGACTCCAGCTTCA
Mouse *Batf3* R	GAGCTGCGTTCTGTTTCTCC
Mouse *Il9* F	CTCTCCGTCCCAACTGATGA
Mouse *Il9* R	GGTCTGGTTGCATGGCTTTT
Mouse *Actb* F	GATTACTGCTCTGGCTCCTAGC
Mouse *Actb* R	GACTCATCGTACTCCTGCTTGC
Mouse *Tnf* F	CAGGCGGTGCCTATGTCTC
Mouse *Tnf* R	CGATCACCCCGAAGTTCAGTAG
