# Supplementary figures and images for: A fungi-derived cyclic peptide enhances Th9-mediated antitumor immunity by targeting ZAP70 and SREBP1
Source: J Clin Invest. 2025 Dec 9;136(3):e196907. doi: 10.1172/JCI196907 (PMC12867150; doi:10.1172/JCI196907)

Figure 5C

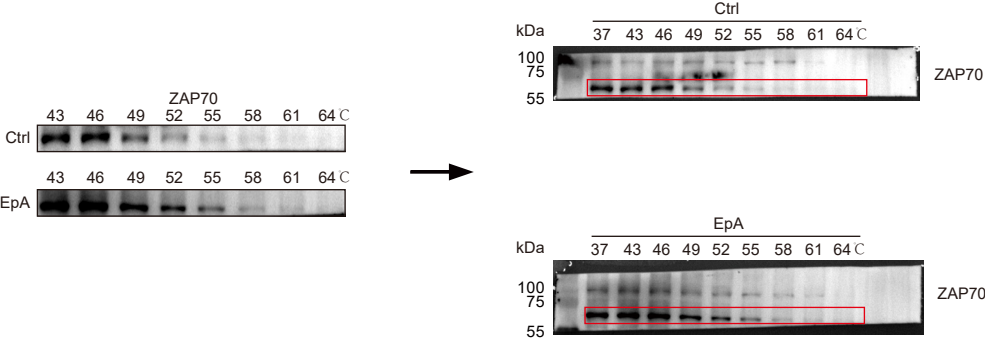

Figure 5G

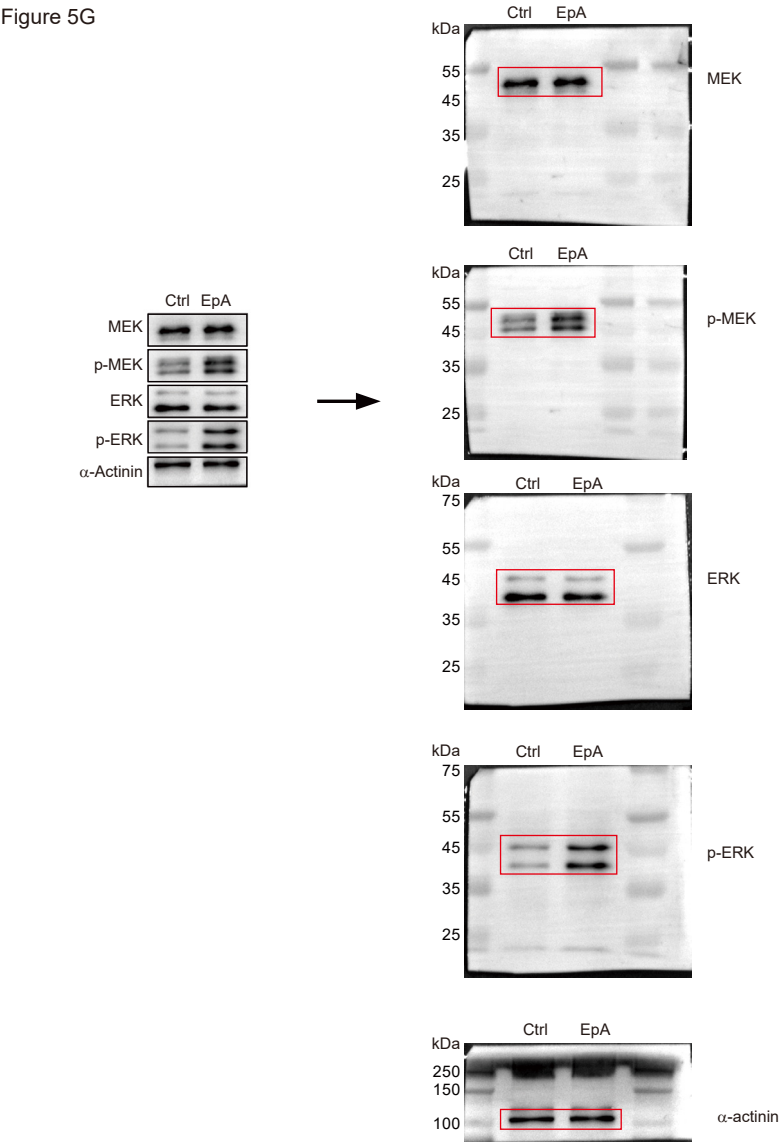

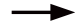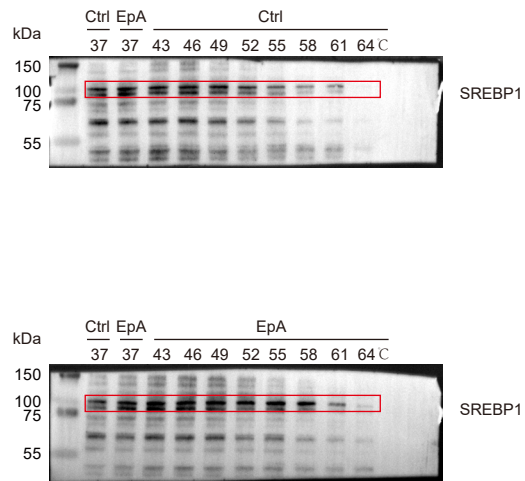

Supplementary Figure 7A

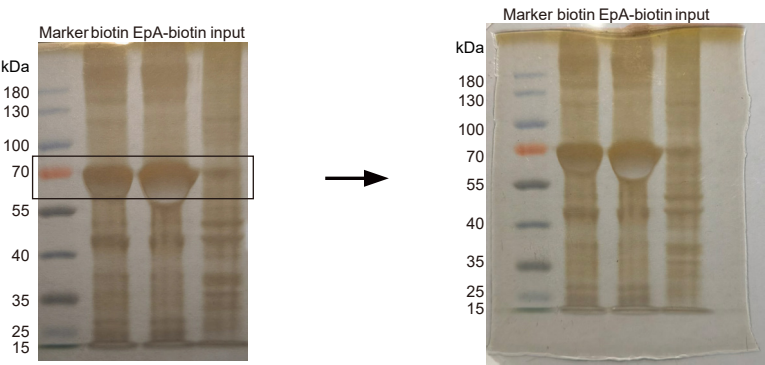

Supplementary Figure 7D

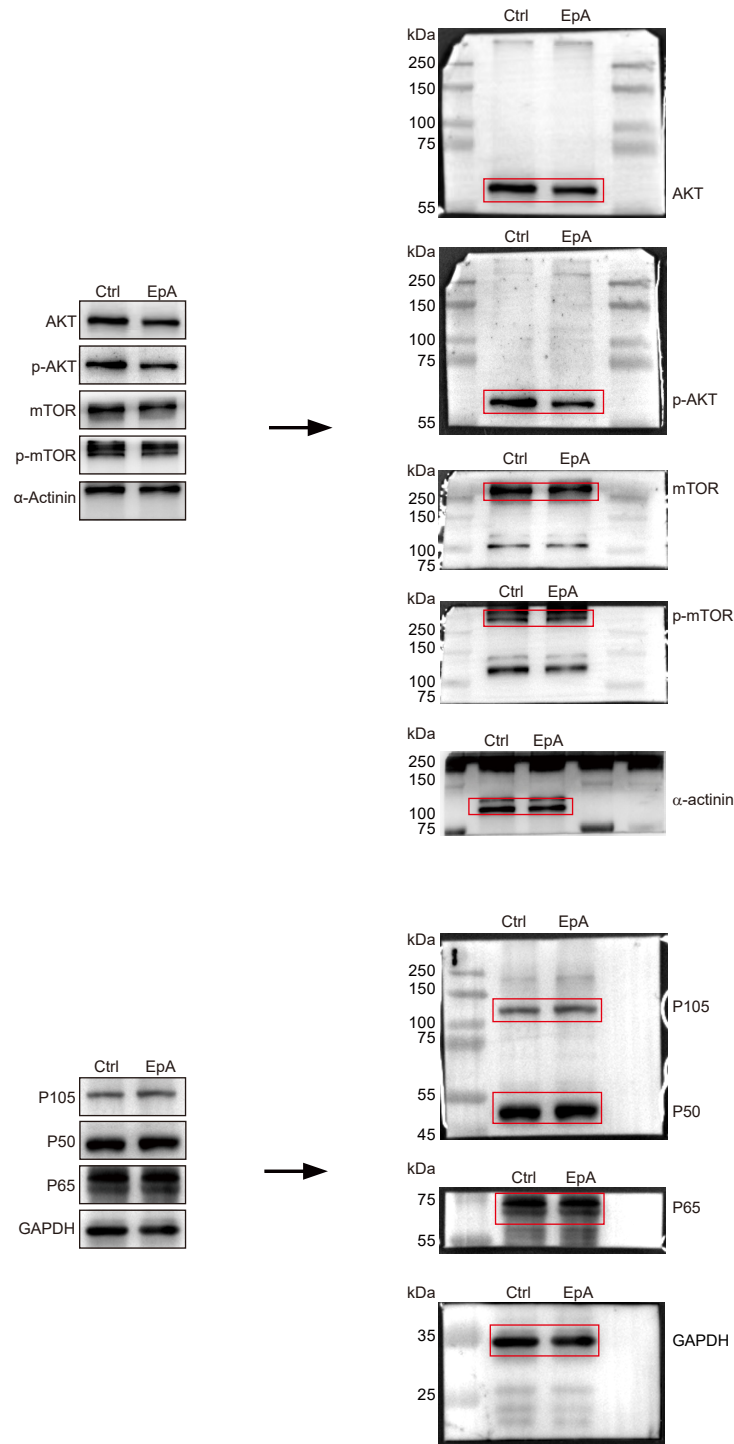

Supplement: Unedited blot and gel images [file jci-136-196907-s132.pdf]
